# Brain Network Topology and Structural–Functional Connectivity Coupling Mediate the Association Between Gut Microbiota and Cognition

**DOI:** 10.3389/fnins.2022.814477

**Published:** 2022-03-29

**Authors:** Shujun Zhang, Xiaotao Xu, Qian Li, Jingyao Chen, Siyu Liu, Wenming Zhao, Huanhuan Cai, Jiajia Zhu, Yongqiang Yu

**Affiliations:** ^1^Department of Radiology, The First Affiliated Hospital of Anhui Medical University, Hefei, China; ^2^Research Center of Clinical Medical Imaging, Hefei, China; ^3^Anhui Provincial Institute of Translational Medicine, Hefei, China; ^4^Department of Radiology, The Fourth Affiliated Hospital of Anhui Medical University, Hefei, China; ^5^Department of Radiology, Chaohu Hospital of Anhui Medical University, Hefei, China

**Keywords:** gut microbiota, cognition, structural connectivity, functional connectivity, topological property, coupling

## Abstract

Increasing evidence indicates that gut microbiota can influence cognition *via* the gut–brain axis, and brain networks play a critical role during the process. However, little is known about how brain network topology and structural–functional connectivity (SC–FC) coupling contribute to gut microbiota-related cognition. Fecal samples were collected from 157 healthy young adults, and 16S amplicon sequencing was used to assess gut diversity and enterotypes. Topological properties of brain structural and functional networks were acquired by diffusion tensor imaging (DTI) and resting-state functional magnetic resonance imaging (fMRI data), and SC–FC coupling was further calculated. 3-Back, digit span, and Go/No-Go tasks were employed to assess cognition. Then, we tested for potential associations between gut microbiota, complex brain networks, and cognition. The results showed that gut microbiota could affect the global and regional topological properties of structural networks as well as node properties of functional networks. It is worthy of note that causal mediation analysis further validated that gut microbial diversity and enterotypes indirectly influence cognitive performance by mediating the small-worldness (*Gamma* and *Sigma*) of structural networks and some nodal metrics of functional networks (mainly distributed in the cingulate gyri and temporal lobe). Moreover, gut microbes could affect the degree of SC–FC coupling in the inferior occipital gyrus, fusiform gyrus, and medial superior frontal gyrus, which in turn influence cognition. Our findings revealed novel insights, which are essential to provide the foundation for previously unexplored network mechanisms in understanding cognitive impairment, particularly with respect to how brain connectivity participates in the complex crosstalk between gut microbiota and cognition.

## Introduction

The scientific study of the microbiota–gut–brain axis (MGB) has provided a wealth of robust generalizations about the influence of gut microbes on the brain and behavior (Johnson and Foster, 2018; Sherwin et al., 2019), particularly with respect to cognitive function (Gareau, 2016; Sarkar et al., 2018). Cognitive impairment has been identified in numerous diseases accompanied by changes in gut microbe structure and metabolic activity, such as irritable bowel syndrome (IBS), inflammatory bowel diseases, obesity, major depressive disorder, and autism spectrum disorder (Gareau, 2016; Sarkar et al., 2018), highlighting the importance of characterizing the mechanism of gut microbiota-related cognition. There have been recent attempts to unpack the inner relationship between gut microbiota and cognition with the application of advanced neuroimaging and microbiome sequencing techniques. For example, using resting-state functional magnetic resonance imaging (rs-fMRI), Liu et al. demonstrated a specific gut microbiota–intrinsic brain activity–cognitive function interaction pattern in patients with amnestic mild cognitive impairment (Liu et al., 2021). A cross-sectional study characterized the relationship between the gut microbiome and mild cognitive impairment through the combined use of MRI and 16S ribosomal RNA high-throughput sequencing (Saji et al., 2019). In addition, some clinical studies have shown that gut microbiota can affect cognitive development including functional brain connectivity and brain structure in infants (Carlson et al., 2018; Gao et al., 2019; Kelsey et al., 2021).

Recent neuroimaging investigations have identified that brain networks may play a critical mediating role between gut microbiota and cognition. Previous correlational studies revealed that gut microbiota alterations caused default mode network (DMN) dysfunction by increasing systemic inflammation and thereby impairing cognition in patients with end-stage renal disease (Zheng et al., 2020). Some longitudinal studies demonstrated that multi-strain probiotic administration reduced depression and improved emotional attention by affecting resting-state functional connectivity (FC) in healthy volunteers (Tillisch et al., 2013; Bagga et al., 2019). Ahluwalia and colleagues indicated that a significant improvement in cognition, including working memory and inhibitory control, through modulation of frontoparietal and subcortical activation and connectivity was seen after gut-specific antibiotic therapy in hepatic encephalopathy (Ahluwalia et al., 2014). Importantly, our previous study also provided evidence that large-scale intra- and inter-network FC mediate the associations of gut microbiota with cognitive performance (Cai et al., 2021). In addition to resting-state FC, structural brain connectivity also mediates behavioral alterations, including cognition, not only in intestinal diseases such as Crohn’s disease (Thomann et al., 2019) and ulcerative colitis (Turkiewicz et al., 2021) but also in extraintestinal diseases such as Alzheimer’s disease (Pereira et al., 2016), schizophrenia (Zhang et al., 2012), and epilepsy (Bernhardt et al., 2011). Although these studies reveal insights into the complex relationship between gut microbes, brain networks, and cognition, there remains insufficient granularity regarding the network architecture and its elements and connections.

Both the brain’s structural and functional systems have features of complex networks (such as local and global efficiency, small-worldness, and node properties), which can be analyzed using a graph theoretical approach. Graph theory provides a theoretical framework in which the topology of complex brain networks can be examined, which can reveal important information about both the local and global organization of brain networks. Moreover, within this framework, nodes (i.e., brain regions) are characterized by measures that quantify their contribution to the anatomical and functional integrity and information flow in the whole brain network (Wang et al., 2014). Recently, how the correlation between structural connectivity (SC) and FC affects human cognition and behavior has received increasing attention. Thus, the concept of structural–functional connectivity coupling (SC–FC) was proposed to predict FC from SC (Honey et al., 2009), which represents the pairwise relationship between the structural and functional networks. Generated multimodality neuroimages may also be more sensitive in detecting brain alterations than any single modality (Bullmore and Sporns, 2009; Honey et al., 2009, 2010; Hagmann et al., 2010; Park and Friston, 2013; Palop and Mucke, 2016; Suarez et al., 2020). In addition, SC–FC coupling allows the characterization of functional dynamics of the brain in terms of spatial topology (Greicius et al., 2009; Honey et al., 2009). Changes in SC–FC coupling occur during brain development (Baum et al., 2020) but also in neuropsychiatric diseases, such as epilepsy (Zhang Z. et al., 2011), schizophrenia (Sun et al., 2017), bipolar disorder (Zhang et al., 2019), Parkinson’s disease (Zarkali et al., 2021), and attention-deficit hyperactivity disorder (Hearne et al., 2019), and may be particularly relevant to cognition: individual differences in coupling reflect differences in cognition (Medaglia et al., 2018; Zimmermann et al., 2018), and SC–FC decoupling is associated with cognitive impairment and Alzheimer’s disease (Qian et al., 2015; Wang et al., 2018; Cao et al., 2020).

However, although the relationship between complex brain networks and cognition has been previously reported in numerous intestinal and extraintestinal diseases, little is known about how the gut microbiome impacts cognition such as the topological properties of brain networks and SC–FC coupling in healthy young adults. Research on the relationships between gut microbiota and complex brain networks might contribute to an understanding of microbiota specialization in functional dynamics of the brain and the mechanisms associated with cognitive ability.

**FIGURE 1:**
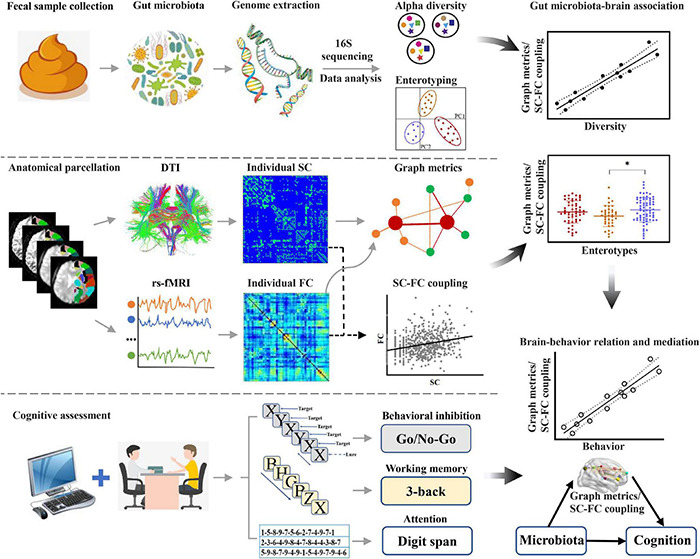
Flowchart of the study design. DTI, diffusion tensor imaging; FC, functional connectivity; rs-fMRI, resting-state functional magnetic resonance imaging; SC, structural connectivity.

In this exploratory study, we performed advanced brain network analysis using graph theory, focusing on the associations of gut microbiota with topological properties of brain networks and SC–FC coupling, as well as how the gut microbiota affects cognition by these brain network metrics. 16S rRNA gene amplicon sequencing was used to assess gut microbial diversity and enterotypes. Topological properties of brain structural and functional networks were acquired by diffusion tensor imaging (DTI) and rs-fMRI data, and SC–FC coupling was further calculated. A set of neuropsychological experimental paradigms (i.e., 3-back, digit span, and Go/No-Go tasks) were employed to assess cognition, including working memory, attention, and behavioral inhibition. Based on a combined analysis of these data, the objectives of this investigation were threefold. First, we attempted to assess the associations of gut microbial diversity and enterotypes with the topological properties of brain networks and SC–FC coupling. Second, we aimed to investigate the potential associations of gut microbiota-linked brain network metrics with cognitive functions. Finally, we sought to establish the mediative role of these identified brain network metrics in accounting for the relationship between gut microbiota and cognition. A systematic flowchart of the study design is shown in Figure 1. We hypothesize that gut microbiota composition is linked to individual variability in topological properties of brain networks and SC–FC coupling, which in turn mediate cognitive performance.

## Materials and Methods

### Participants

A total of 157 healthy young adults were recruited by advertisement. All participants met the inclusion criteria of Chinese Han, right handedness, and within a restricted age range of 18–30 years. Exclusion criteria included neuropsychiatric or severe somatic disorder, a history of alcohol or drug abuse, regular smoker, current medication (e.g., antibiotics or sedative hypnotics) within a month, pregnancy, MRI contraindications, and a family history of psychiatric illness among first-degree relatives. The MINI-International Neuropsychiatric Interview (M.I.N.I.) and Alcohol Use Disorders Identification Test (AUDIT) were used in the process of excluding participants. This study was approved by the ethics committee of The First Affiliated Hospital of Anhui Medical University. Written informed consent was obtained from all participants after they had been given a complete description of the study.

### Cognition Assessment

Three tasks were used to assess cognitive function in our study. The letter 3-back and the Go/No-Go tasks were conducted on a computer to assess working memory (Owen et al., 2005) and the ability of behavioral inhibition (Kaufman et al., 2003), respectively. We also adopted digit span tasks to evaluate attention (Groth-Marnat and Baker, 2003). Full information is described in detail in Supplementary Material.

### MRI Data Acquisition and Preprocessing

High-resolution 3D T1-weighted structural images, DTI data, and resting-state blood-oxygen-level-dependent (BOLD) fMRI were obtained using a 3.0-Tesla MR system (Discovery MR750w, General Electric, Milwaukee, WI, United States) with a 24-channel head coil. The software packages FMRIB Software Library (FSL),^1^ Diffusion Toolkit (DTK),^2^ and Pipeline for Analyzing braiN Diffusion imAges (PANDA)^3^ were used for the DTI preprocessing steps. Resting-state BOLD data were preprocessed using SPM12 and Data Processing and Analysis for Brain Imaging (DPABI).^4^ The details are described in Supplementary Methods.

### Structural and Functional Brain Network Construction

Whole-brain SC and FC matrices for each individual subject were reconstructed, consisting of 90 cortical areas, reflecting a subdivision of the Automated Anatomical Labeling (AAL) atlas (Tzourio-Mazoyer et al., 2002). For structural brain network, we reconstructed streamlines between these areas and calculated the number of fibers (with end points located in both nodes during the fiber tracking) between any pairs of nodes, resulting in a 90 × 90 fiber number (FN) matrix for each subject. Two nodes were considered structurally connected when the FN between them was at least 3 (for details, see Supplementary Methods). For resting-state data, regional time series were calculated as the mean across voxels within each region included in the brain parcellation. For each individual, Pearson’s correlations were calculated between the time series of all regions to calculate FC. Finally, a Fisher z-transformation was applied to the FC matrices (for details, see Supplementary Methods).

### Network Topological Metrics

Graph theoretical analyses were carried out on structural and functional network using GRETNA software^5^ (Wang et al., 2015). We calculated both global and regional topological metrics for each individual. The global network metrics included global efficiency (E_glob_, the efficiency of information transfer through the entire graph), local efficiency (E_loc_, the average efficiency of information transfer over a node’s direct neighbors), and five small-world property metrics (Watts and Strogatz, 1998). The small-world property metrics included the clustering coefficient (Cp, the average inter-connectedness of a node’s direct neighbors), characteristic path length (Lp, the average shortest path length between any pairs of nodes), normalized clustering coefficient (*Gamma*, the ratio of clustering coefficients between real and random networks), normalized characteristic path length (*Lambda*, the ratio of the characteristic path lengths between real and random networks), and small-worldness property (*Sigma* = *Gamma*/*Lambda*, a scalar quantitative measurement of the small-worldness of a network) (Supplementary Figure 1). For the regional network metrics, we evaluated nodal degree centrality (D_nodal_), nodal efficiency (E_nodal_), and nodal betweenness (B_nodal_) (Rubinov and Sporns, 2010). The number of random networks was set as 100. Binary graph methods for global and regional topological metrics were used, as they were computationally straightforward and provided for simpler interpretation (for details, see Supplementary Methods). For FC matrix, we applied a range of sparsity thresholds (range of 0.10–0.34 with an interval of 0.01) to ensure the generated networks were estimable for small-worldness and had sparse properties with the minimum possible number of spurious edges (Zhang J. et al., 2011; Lei et al., 2015; Suo et al., 2015). The area under the curve (AUC) for each network metric was calculated for subsequent analysis, which provided a summarized scalar for the topological characterization of brain networks. Since the integrated AUC metric is independent of a single threshold selection and is sensitive to topological alterations of brain disorders, it has been extensively used in brain network studies (He et al., 2009; Wang et al., 2009; Zhang J. et al., 2011; Lei et al., 2015; Suo et al., 2015).

### Structural–Functional Connectivity Coupling

Structural–functional connectivity coupling was assessed by the correlation coefficient between strengths of the structural and functional networks. To this end, non-zero SC edges were extracted to form a vector of SC values, which was further normalized using a log transformation (Collin et al., 2017; Hearne et al., 2019; Koubiyr et al., 2019). The resulting SC values were correlated with corresponding FC values (i.e., the same edges) in the level of brain regions. Finally, this analysis produced 90 Pearson’s r values representing the SC–FC coupling of 90 cortical areas for each individual subject.

### Fecal Samples Collection and Gut Microbiota Analysis

Microbial genome DNA was extracted from the fecal samples using a QIAamp DNA Stool Mini Kit (Qiagen Inc., Hilden, Germany). The V4 region of 16S ribosomal RNA (rRNA) gene was amplified. The qualified amplicon mixture was then sequenced on the MiSeq platform with the PE250 sequencing strategy. Alpha diversity was assessed using the species diversity indices (including Shannon and Simpson indices) (Table 1; Faith, 1992; Keylock, 2005), which were calculated by MOTHUR (v1.31.2) (Schloss et al., 2009) and QIIME (v1.8.0) (Caporaso et al., 2010) at the operational taxonomic unit (OTU) level. Shannon index measures the average degree of uncertainty in predicting where individual species chosen at random will belong, which increases as the number of species increases and as the distribution of individuals among the species becomes even (Lemos et al., 2011; Kim et al., 2017). Simpson index indicates the species dominance and reflects the probability of two individuals that belong to the same species being randomly chosen, which varies from 0 to 1, and the index decreases as the diversity increases (Kim et al., 2017). Shannon and Simpson indices reflect both species richness and species evenness. Sample enterotyping was performed based on OTU-derived genus abundance matrix as described in the original publication (for details, see Supplementary Methods). All samples were clustered into three well-matched enterotypes (Supplementary Figure 2 and Supplementary Table 1). Prevotella, Ruminococcaceae, and Bacteroides genera were considered as enterotype identifiers (P-, R-, and B-enterotypes) as they showed the largest variation in abundance, coinciding with prior studies (Arumugam et al., 2011; Falony et al., 2016; Vieira-Silva et al., 2016). In addition, the species accumulation curves were plotted in Supplementary Figure 3, which indicated that the sampling amount was sufficient.

**TABLE 1:** Demographic, gut microbial, and behavioral characteristics of the participants.

Characteristics	Mean ± SD	Range
Gender (female/male)	77/80	–
Age (years)	22.32 ± 2.42	18–28
Education (years)	15.78 ± 1.92	12–20
BMI (kg/m^2^)	21.44 ± 3.20	15.42–36.99
FD (mm)	0.12 ± 0.05	0.04–0.40
**Alpha diversity**		
Shannon index	3.06 ± 0.52	1.61–3.97
Simpson index	0.13 ± 0.09	0.03–0.51
**3-Back task performance**		
Accuracy	0.72 ± 0.16	0.15–0.98
Reaction time (ms)	768.87 ± 175.24	230.23–1,179.93
**Digit span task performance**		
Digit span forward	8.49 ± 1.29	5–13
Digit span backward	6.57 ± 1.54	3–10
**Go/No-Go task performance**		
Acc_No-Go	0.59 ± 0.19	0.05–1.00
Acc_Go	0.95 ± 0.10	0.47–1.00
RT_Go (ms)	432.83 ± 69.57	256.73–591.64

*Acc_No-Go, accuracy in “No-Go” conditions; Acc_Go, accuracy in “Go” conditions; BMI, body mass index; FD, frame-wise displacement; RT_Go, mean reaction time of correct responses in “Go” conditions.*

### Statistical Analysis

The statistical descriptive analyses of demographic, gut microbial, and behavioral data were conducted using the SPSS 23.0 software package (SPSS, Chicago, IL, United States). We adopted a multi-stage approach to analyze the data of gut microbiota (alpha diversity and enterotypes), network metrics (global and regional topological properties and SC–FC coupling), and cognitive performance (working memory, behavior inhibition, and attention). First, we tested for gut microbiota–brain network associations by performing partial correlation analyses between alpha diversity and network metrics with age, sex, and body mass index (BMI) as nuisance covariates [*p* < 0.05, false discovery rate (FDR) corrected]. For the metrics related to FC, frame-wise displacement (FD) was added as a covariate. Second, group comparisons of network metrics across enterotypes were performed using one-way analysis of variance (ANOVA) followed by *post-hoc* Bonferroni test (*p* < 0.05). Third, once significant correlations or inter-group differences were identified in any network metrics, we further examined their associations with cognitive functions using partial correlations adjusting for age, sex, BMI, and educational level. The metrics related to FC had additional adjustment for FD. Finally, we performed mediation analysis using the PROCESS macro^6^ (Hayes, 2009) to further elucidate the relationship among gut microbiota, brain network metrics, and cognition. In the mediation models, all paths were reported as unstandardized ordinary least squares regression coefficients, namely, total effect of X on Y (c) = indirect effect of X on Y through M (a × b) + direct effect of X on Y (c’). The significance analysis was based on 10,000 bootstrap realizations, and a significant indirect effect is indicated when the bootstrap 95% confidence interval (CI) does not include 0. In the mediation analysis, only variables that showed a significant correlation with others were considered as independent (gut microbiota), dependent (cognitive functions), or mediating (brain network metrics) variables. Age, sex, BMI, and educational level were considered as nuisance variables. The metrics related to FC had additional adjustment for FD.

## Results

### Demographic, Cognitive, and Gut Microbial Characteristics

The demographic, cognitive, and gut microbial diversity of the participants are listed in Table 1. In addition, information on the three enterotypes (including Prevotella, Ruminococcaceae, and Bacteroides genera) is listed in Supplementary Figure 2 and Supplementary Table 1.

### The Associations Between Gut Microbiota, Structural Brain Networks, and Cognition

**FIGURE 2:**
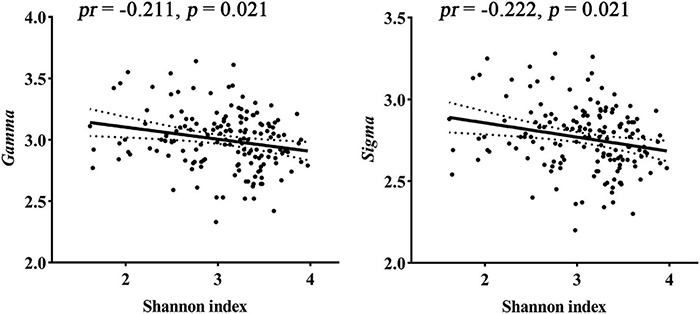
Correlations between gut microbial diversity and small-worldness parameters of SC. *pr*, partial correlation coefficient; SC, structural connectivity.

For global topological metrics, structural networks exhibited higher clustering coefficients (i.e., *Gamma*; mean = 2.998, *SD* = 0.238) but almost identical characteristic path lengths (i.e., *Lambda*; mean = 1.084, *SD* = 0.011) relative to comparable random networks, which indicates that the structural network showed a typical small-world topology (i.e., *Sigma* > 1). Correlation analysis showed that the Shannon index was negatively correlated with *Gamma* (*pr* = −0.211, *p* = 0.021) and *Sigma* (*pr* = −0.222, *p* = 0.021, FDR corrected; Figure 2). There were no significant inter-group differences in global topological metrics among the three enterotypes.

**FIGURE 3:**
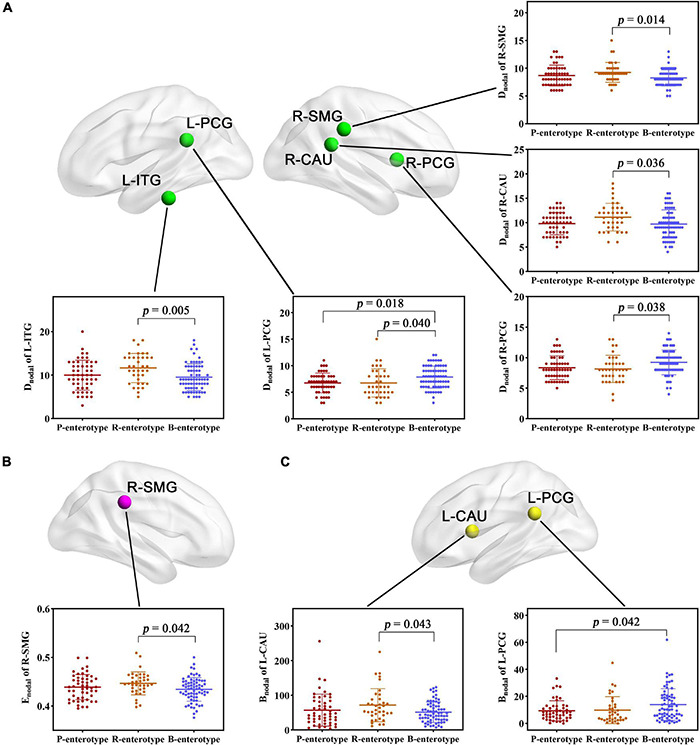
**(A–C)** Inter-group comparisons in regional topological metrics of SC across enterotypes. Spatial maps show the significantly different nodes (i.e., brain regions). B_nodal_, nodal betweenness; CAU, right caudate nucleus; D_nodal_, nodal degree centrality; E_nodal_, nodal efficiency; ITG, inferior temporal gyrus; L, left; PCG, posterior cingulate gyrus; R, right; SMG, supramarginal gyrus; SC, structural connectivity.

For regional topological metrics, there was no significant correlation between alpha diversity and regional topological metrics of the structural network. However, one-way ANOVA found that some D_nodal_, E_nodal_, and B_nodal_ of structural networks exhibited significant inter-group differences across the three enterotypes (*p* < 0.05, *post-hoc* Bonferroni test; Figure 3). The D_nodal_ of the left posterior cingulate gyrus (L-PCG), left inferior temporal gyrus (L-ITG), right posterior cingulate gyrus (R-PCG), right supramarginal gyrus (R-SMG), and right caudate nucleus (R-CAU) (Figure 3A); the E_nodal_ of the R-SMG (Figure 3B); and the B_nodal_ of the L-PCG and left caudate nucleus (L-CAU) (Figure 3C) showed significant inter-group differences among the three enterotypes (all *p* < 0.05, *post-hoc* Bonferroni test; Figure 3).

**FIGURE 4:**
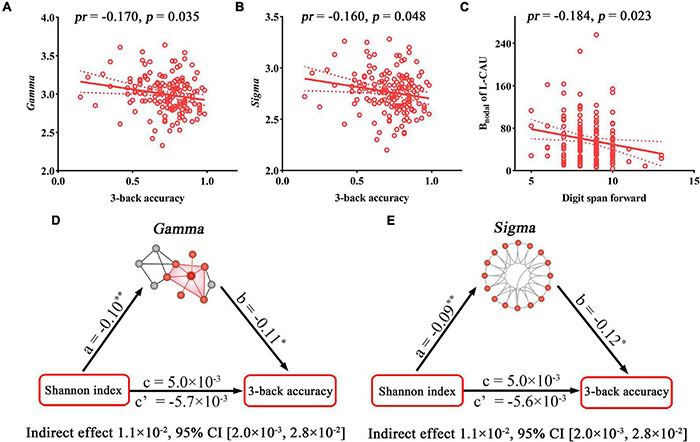
Correlations and mediations between topological properties of SC, gut microbiota, and cognition. **(A–C)** Scatter plots of the correlations between topological metrics of SC and cognition. **(D,E)** The mediation analyses between gut microbial diversity (X) and 3-back accuracy (Y), with small-worldness parameters of SC as the mediator (M). Path coefficients with *p*-values (*p < 0.05 and **p < 0.01, respectively). B_nodal_, nodal betweenness; CI, confidence interval; *pr*, partial correlation coefficient; SC, structural connectivity.

The relationships between cognition and network metrics that exhibited significant correlations or inter-group differences were further investigated. 3-Back accuracy was found to be negatively correlated with *Gamma* (*pr* = −0.170, *p* = 0.035) and *Sigma* (*pr* = −0.160, *p* = 0.048) (Figures 4A,B), which are related to the Shannon index. Digit span forward showed a significant negative correlation with the B_nodal_ of the L-CAU (*pr* = −0.184, *p* = 0.023) (Figure 4C), which exhibited inter-group differences. Further mediation analyses revealed that both *Gamma* and *Sigma* mediated the relationships between the Shannon index and 3-back accuracy (Figures 4D,E).

### The Associations Between Gut Microbiota, Functional Brain Networks, and Cognition

For global topological metrics, in the defined sparsity threshold range, functional brain networks also exhibited higher clustering coefficients (i.e., *Gamma* > 1) but almost identical characteristic path lengths (i.e., *Lambda* ≈ 1) relative to comparable random networks, which indicates that functional brain networks showed a typical small-world topology (i.e., *Sigma* > 1) (Supplementary Figure 4). Although functional networks satisfied small-world topology, no significant relationship of gut microbiota with global topological metrics of the functional network was found.

**FIGURE 5:**
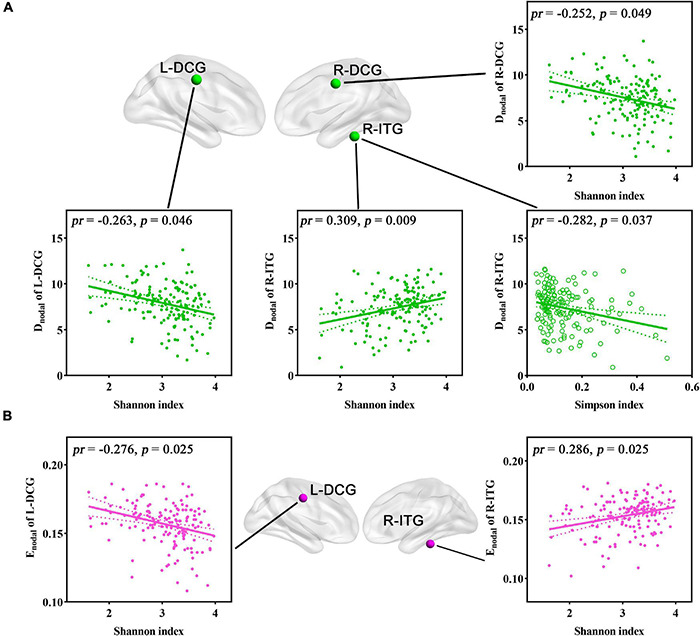
**(A,B)** Correlations between gut microbial diversity and regional topological metrics of FC. Spatial maps show the significantly related nodes (i.e., brain regions). DCG, median cingulate and paracingulate gyri; D_nodal_, nodal degree centrality; E_nodal_, nodal efficiency; FC, functional connectivity; ITG, inferior temporal gyrus; L, left; *pr*, partial correlation coefficient; R, right.

**FIGURE 6:**
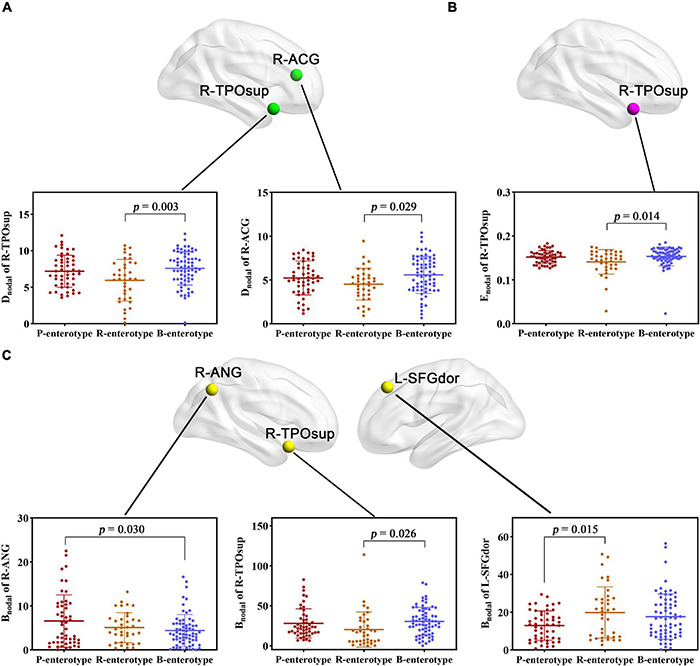
**(A–C)** Inter-group comparisons in regional topological metrics of FC across enterotypes. Spatial maps show the significantly different nodes (i.e., brain regions). ACG, anterior cingulate and paracingulate gyri; ANG, angular gyrus; B_nodal_, nodal betweenness; D_nodal_, nodal degree centrality; E_nodal_, nodal efficiency; L, left; R, right; SFGdor, dorsolateral superior frontal gyrus; TPOsup, temporal pole: superior temporal gyrus.

For regional topological metrics, correlation analyses revealed significant correlations between alpha diversity (Shannon and Simpson indices) and regional topological metrics of the functional network (*p* < 0.05, FDR corrected; Figure 5). The Shannon index was negatively correlated with the D_nodal_ of the left median cingulate and paracingulate gyri (L-DCG, *pr* = −0.263, *p* = 0.046) and right median cingulate and paracingulate gyri (R-DCG) (*pr* = −0.252, *p* = 0.049) and positively correlated with the D_nodal_ of the right inferior temporal gyrus (R-ITG, *pr* = 0.309, *p* = 0.009). The Simpson index was negatively correlated with the D_nodal_ of the R-ITG (*pr* = −0.282, *p* = 0.037, FDR corrected; Figure 5A). Moreover, the Shannon index was negatively correlated with the E_nodal_ of the L-DCG (*pr* = −0.276, *p* = 0.025) and positively correlated with the E_nodal_ of the R-ITG (*pr* = 0.286, *p* = 0.025, FDR corrected; Figure 5B). In addition, one-way ANOVA found that some D_nodal_, E_nodal_, and B_nodal_ of functional networks exhibited significant inter-group differences across the three enterotypes (*p* < 0.05, *post-hoc* Bonferroni test; Figure 6). The D_nodal_ of the right anterior cingulate and paracingulate gyri (R-ACG) and the right temporal pole: superior temporal gyrus (R-TPOsup) (Figure 6A); the E_nodal_ of the R-TPOsup (Figure 6B); and the B_nodal_ of the right angular gyrus (R-ANG), R-TPOsup, and left dorsolateral superior frontal gyrus (L-SFGdor) (Figure 6C) showed significant inter-group differences among the three enterotypes (all *p* < 0.05, *post-hoc* Bonferroni test; Figure 6).

**FIGURE 7:**
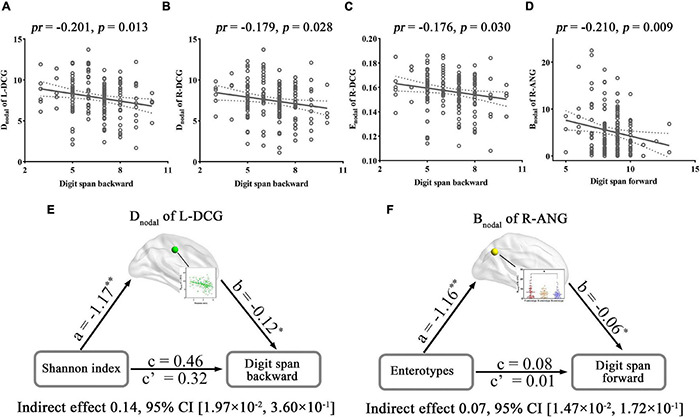
Correlations and mediations between regional topological properties of FC, gut microbiota, and cognition. **(A–D)** Scatter plots of the correlations between topological metrics of FC and cognition. **(E,F)** The mediation analyses between gut microbiota (X) and digit span forward and backward (Y), with nodal parameters of FC as the mediator (M). Path coefficients with *p*-values (*p < 0.05 and ^**^p < 0.01, respectively). ANG, angular gyrus; B_nodal_, nodal betweenness; CI, confidence interval; D_nodal_, nodal degree centrality; DCG, median cingulate and paracingulate gyri; E_nodal_, nodal efficiency; FC, functional connectivity; L, left; *pr*, partial correlation coefficient; R, right.

The regional metrics that exhibited significant correlations or inter-group differences with gut microbiota and their associations with cognition were further examined. For alpha diversity-related nodal metrics, digit span backward was found to be negatively correlated with the D_nodal_ of the L-DCG (*pr* = −0.201, *p* = 0.013) (Figure 7A) and R-DCG (*pr* = −0.179, *p* = 0.028) (Figure 7B) and the E_nodal_ of the R-DCG (*pr* = −0.176, *p* = 0.030) (Figure 7C). In addition, digit span forward showed a significant negative correlation with the B_nodal_ of the R-ANG (*pr* = −0.210, *p* = 0.009) (Figure 7D), which exhibited inter-group differences. Further mediation analyses revealed that the D_nodal_ of the L-DCG mediated the relationships between the Shannon index and digit span backward (Figure 7E), and the B_nodal_ of the R-ANG mediated the relationships between enterotypes and digit span forward (Figure 7F).

### The Associations Between Gut Microbiota, Structural–Functional Connectivity Coupling, and Cognition

**FIGURE 8:**
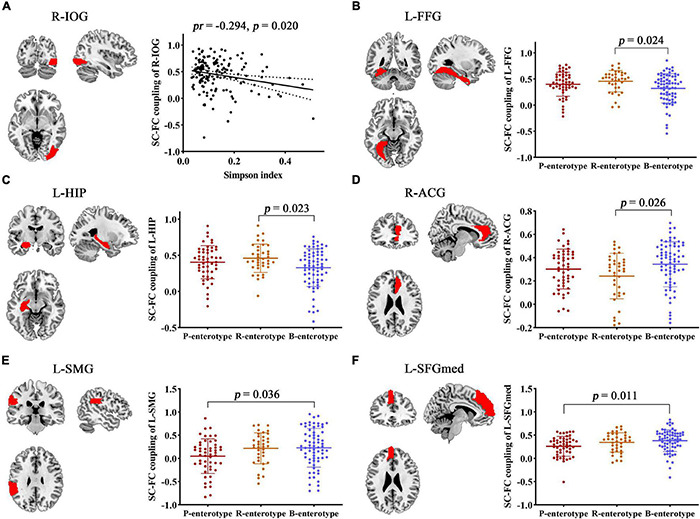
Correlation and inter-group comparisons between SC–FC coupling, gut microbial diversity, and enterotypes. Spatial maps show the significantly related or different brain regions. **(A)** Scatter plots of the correlations between gut microbial diversity and SC–FC coupling. **(B–F)** Inter-group comparisons in SC–FC coupling across enterotypes. ACG, anterior cingulate and paracingulate gyri; FC, functional connectivity; FFG, fusiform gyrus; HIP, hippocampus; IOG, inferior occipital gyrus; L, left; *pr*, partial correlation coefficient; R, right; SC, structural connectivity; SMG, supramarginal gyrus; SFGmed, medial superior frontal gyrus.

Correlation analyses revealed significant correlations between the Simpson index and the SC–FC coupling of the right inferior occipital gyrus (R-IOG, *pr* = −0.249, *p* = 0.020, FDR corrected; Figure 8A). One-way ANOVA found that the SC–FC coupling of the left fusiform gyrus (L-FFG), left hippocampus (L-HIP), R-ACG, left supramarginal gyrus (L-SMG), and left medial superior frontal gyrus (L-SFGmed) exhibited significant inter-group differences among the three enterotypes (all *p* < 0.05, *post-hoc* Bonferroni test; Figures 8B–F).

**FIGURE 9:**
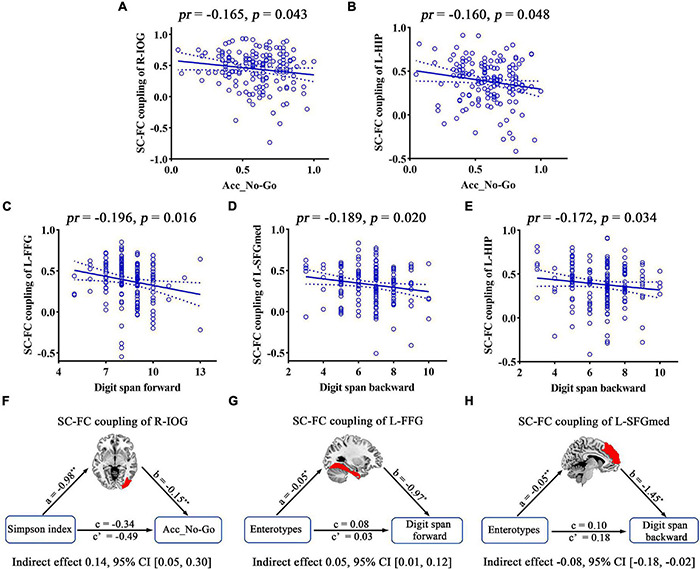
Correlations and mediations between SC–FC coupling, gut microbiota, and cognition. **(A–E)** Scatter plots of the correlations between SC–FC coupling and cognition. **(F–H)** The mediation analyses between gut microbiota (X) and cognition (Y), with SC–FC coupling as the mediator (M). Path coefficients with *p*-values (*p < 0.05 and **p < 0.01, respectively). Acc_No-Go, the accuracy in “No-Go” conditions; CI, confidence interval; FC, functional connectivity; FFG, fusiform gyrus; HIP, hippocampus; IOG, inferior occipital gyrus; L, left; *pr*, partial correlation coefficient; R, right; SC, structural connectivity; SFGmed, medial superior frontal gyrus.

Structural–functional connectivity coupling in multiple brain regions was affected by gut microbiota, and its associations with cognition were further investigated. Accuracy in “No-Go” conditions (Acc_No-Go) was found to be negatively correlated with the SC–FC coupling of the R-IOG related to the Simpson index (*pr* = −0.165, *p* = 0.043) (Figure 9A). Furthermore, the metrics that exhibited inter-group differences were also found to be significantly correlated with cognitive performance. Acc_No-Go was negatively correlated with the SC–FC coupling of the L-HIP (*pr* = −0.160, *p* = 0.048) (Figure 9B), digit span forward was negatively correlated with the SC–FC coupling of the L-FFG (*pr* = −0.196, *p* = 0.016) (Figure 9C), and digit span backward was negatively correlated with the SC–FC coupling of the L-SFGmed (*pr* = −0.189, *p* = 0.020) (Figure 9D) and L-HIP (*pr* = −0.172, *p* = 0.034) (Figure 9E). Further mediation analyses revealed that the SC–FC coupling of the R-IOG mediated the relationships between the Simpson index and Acc_No-Go (Figure 9F), the SC–FC coupling of the L-FFG mediated the relationships between enterotypes and digit span forward (Figure 9G), and the SC–FC coupling of the L-SFGmed mediated the relationships between enterotypes and digit span backward (Figure 9H).

## Discussion

This study is the first to assess the relationship between the gut microbiome, brain network topological parameters, and cognition in healthy young adults. The current multisite effort yielded three main findings. First, we found that the gut microbiota could influence the global and regional topological properties of structural networks. Further mediation analysis confirmed that global topological metrics (*Gamma and Sigma*) of structure networks mediated the relationships between alpha diversity and working memory. Second, only the node properties of functional networks showed significant correlations and inter-group differences with gut microbiota, which were mainly distributed in the cingulate gyri and temporal lobe. Moreover, some nodal metrics (D_nodal_ of the median cingulate and paracingulate gyri and B_nodal_ of the angular gyrus) could serve as mediators of the associations between gut microbiota and attention. Third, gut microbes could affect the degree of SC–FC coupling in the inferior occipital gyrus, fusiform gyrus, and medial superior frontal gyrus, which in turn influence cognitive performance (including behavior inhibition and attention). We thus conclude that the topological properties of brain networks and SC–FC coupling were affected by gut microbes, which may help to reveal a potential network mechanism of gut microbiota-related cognition.

Both the brain’s structural and functional systems have features of complex networks that strike the best balance between local specialization and global integration, which is supported by the local efficiency and the characteristic path length (Wang et al., 2014). Structural brain networks typically correspond to white matter tracts between pairs of brain regions and provide a neurobiological basis for information transmission between cortical regions (Gong et al., 2009). A previous study reported that inflammatory bowel diseases (including ulcerative colitis and Crohn’s disease) are usually accompanied by changes in gut microbiome structure and metabolic activity (Franzosa et al., 2019). Moreover, altered interactions between gut microbes and the intestinal immune system may be more directly linked to or impact the brain through the gut–brain axis (Franzosa et al., 2019; Zhang et al., 2021). Compared to healthy controls, both ulcerative colitis (Turkiewicz et al., 2021) and Crohn’s disease (Thomann et al., 2019) exhibited abnormal alterations in white matter connectivity and network architecture (such as small−worldness property, nodal degree, and nodal betweenness centrality). It is more important that inflammatory bowel diseases have recently been shown to increase the risk of cognitive decline (Lackner et al., 2018; Zhang et al., 2021). However, the causal relationship between gut microbe–white matter network architecture and cognition has not yet been confirmed. In our study, *Gamma* and *Sigma* of small-world property metrics decreased as gut microbial diversity increased, whereas cognitive performance was improved as *Gamma* and *Sigma* decreased. For the unexpected results of small-world measures, the possible reasons might be that *Gamma* and *Gamma*-driven *Sigma* reflect regional specializations of information processing in the network, which were usually above average in healthy adults (Bassett and Bullmore, 2006; Wang et al., 2019). The increase in intra-regional connection density facilitates information specialization within the specific network, while the sparse inter-regional connections serve as shortcuts to reduce the characteristic path length of the network and enhance global information integration (Liao et al., 2017). Thus, the small-world measures (*Gamma* and *Sigma*) decrease as diversity increases, which might facilitate the balance of informational specialization and globalization of the structural network, which in turn improves cognitive performance. An alternative explanation is that the limited DTI technique (e.g., 64 diffusion directions) and relatively modest sample size might lead to unstable results, which need to be validated in future research. In addition, the D_nodal_, E_nodal_, and B_nodal_ of some brain regions showed significant inter-group differences across the three enterotypes (Prevotella, Ruminococcaceae, and Bacteroides genera). D_nodal_, E_nodal_, and B_nodal_ variously assess the importance of individual nodes; important brain regions usually interact with many other regions, facilitate functional integration, and play a critical role in network resilience to insult. These brain regions that are susceptible to gut microbiota are mainly distributed in the posterior DMN (such as the posterior cingulate gyrus and supramarginal gyrus) and caudate nucleus, which are thought to be involved in complex cognition and are linked to memory or abstract thought (Crespo-Facorro et al., 2007; Muller et al., 2018; Smallwood et al., 2021). More importantly, causal mediation analysis further validated that the changes in complexity and diversity of gut microbes indirectly influence working memory by mediating the alterations in *Sigma* and *Gamma* in our study. Working memory is a higher-order cognitive activity that requires integration and dynamic interaction across various cerebral sites (Li et al., 2009), which supports current findings. Overall, our results, together with prior work, hint at the alterations in topological properties of structural networks, mainly with regard to small-worldness, and could be a potential network mechanism for how gut microbiota affect cognition.

Functional networks were constructed from time series of brain dynamics simulated based on anatomical parcellation, which represent patterns of cross-correlations between BOLD signals estimated from these dynamics and may occur between pairs of anatomically unconnected regions (Zhou et al., 2009; Rubinov and Sporns, 2010). Reports of the relationship between gut microbiota and functional connectivity are more common and prevalent in intestinal and extraintestinal diseases (Tillisch et al., 2013; Ahluwalia et al., 2014; Bagga et al., 2019; Gao et al., 2019; Zheng et al., 2020; Cai et al., 2021). However, most functional connectivity was measured using a hypothesis-driven seed-based approach and independent component analysis (ICA), and only a few studies have used graph theory models to explore the topological properties of intrinsic connectivity networks in disease. For example, Wang and colleagues found that gut microbiota alteration impaired DMN topological properties (including clustering coefficient, local efficiency, *Gamma*, and *Sigma*) by increasing systemic inflammation in end-stage renal disease patients (Wang et al., 2019). Certainly, the occurrence of some diseases is closely correlated with gut microbial alteration, and these diseases show changes in topological parameters of the functional network at the same time, which might provide indirect evidence that gut microbiota can influence the topological properties of functional networks, such as in IBS (Kano et al., 2020) and Crohn’s disease (Kong N. et al., 2021). In this study, we found that some nodal properties of the brain functional network were related to the diversity and enterotypes of the gut microbiota in healthy young adults. Associated nodes were mainly distributed in the brain regions related to high-level cognitive processes. For example, the DCG is related to memory, spatial orientation, and attentional control (Cieslik et al., 2015; Song et al., 2021); the ITG and TPOsup are correlated with visual and language comprehension and emotion regulation (Lin et al., 2020; Herlin et al., 2021); and the ANG is involved in semantic processing, word reading and comprehension, number processing, the DMN, and memory retrieval (Seghier, 2013). Particularly, some regional topology measures of functional networks were negatively correlated with cognition in our study. One possible reason is that the anterior cingulate and part of the paracingulate gyri and angular gyri are traditionally considered to belong to the DMN (Vatansever et al., 2015; Jenkins, 2019; Leng et al., 2020; Peng et al., 2021; Tu et al., 2021). The DMN exhibits high activity at rest (Raichle et al., 2001), which is believed to support internally oriented processes such as recalling autobiographical and episodic memories, envisioning the future, and making social inferences (Fan et al., 2021). During externally directed or attention-demanding tasks, DMN activity is suppressed, and the level of DMN suppression is reported to be associated with task performance (Kelly et al., 2008; Hampson et al., 2010). Therefore, the DMN is also known as the task-negative network. Moreover, the D_nodal_ of L-DCG and B_nodal_ of R-ANG mediated the associations between gut microbiota and cognition. However, no significant correlation was observed between all global topological parameters and gut microbiota in this study, which partly explained a previous study in which global normalized graph measures did not show any significant differences in IBS patients compared to healthy controls (Kano et al., 2020). Taken together, our findings support the notion that the gut microbiome may impact cognition primarily through the regional topological properties of functional networks.

Structural–functional connectivity coupling represents the pairwise relationship between the structural and functional networks and has been widely used in exploring cognitive impairment and Alzheimer’s disease (Qian et al., 2015; Wang et al., 2018; Cao et al., 2020; Koubiyr et al., 2021). However, to the best of our knowledge, no studies on the relationships between gut microbiota, SC–FC coupling, and cognition have been performed to date. The present study provides new evidence for uncovering the correlation between gut microbiota and cognition. We found that gut microbial diversity affects the degree of SC–FC coupling in the R-IOG, which in turn influences cognitive performance. R-IOG was traditionally recognized as the visual cortex, and the prominent R-IOG involvement may be interpreted as the fact that detecting and processing visual stimuli are a prerequisite for Go/No-Go tasks. In addition, SC–FC coupling in multiple brain regions exhibited significant inter-group differences across the three enterotypes. A seminal study revealed that the enterotypes are mostly driven by species composition (Arumugam et al., 2011), which might contribute to explaining neural activity potential variation between the different enterotypes. Surprisingly, we found some unexpected negative correlations between SC–FC measures and cognition. One explanation for this may be that the coupling between regional structural and functional connectivity profiles varied widely across the cortex, and highly conserved sensory areas had relatively strong structure–function coupling, while highly expanded transmodal areas had weaker coupling (Baum et al., 2020). In our study, the regions associated with gut microbes were mainly located in higher brain regions involved in complex activities, such as L-FFG and L-HIP, which need to integrate information across distinct brain modules and may connect to anatomically unconnected regions during tasks. Furthermore, L-HIP and L-SFGmed are located in the DMN (Raichle, 2015), which often show reductions in activity during attention-demanding tasks but increase their activity across multiple forms of intrinsic cognition, many of which are linked to memory or abstract thought (Smallwood et al., 2021). These studies partly account for our findings that the SC–FC coupling of L-SFGmed and FFG mediated the relationships between enterotypes and cognitive performance. Altogether, these findings shed new light on the microbial origins of individual differences in SC–FC coupling and cognition and provide a new perspective for future research on interventions of gut microbiota in mental disease with cognitive decline.

The present study has several limitations that should be acknowledged. First, the cross-sectional design limits our ability to make causal inferences. Future prospective longitudinal studies are needed to resolve the causality of the complex gut microbiota–brain network topological properties–cognition relationship. Second, our study sample was selected from a group of educated young adults, thus limiting the generalizability of the findings. Third, group comparisons analysis in network metrics across enterotypes using one-way ANOVA and *post-hoc* Bonferroni test were performed, but no more rigorous corrections for multiple one-way ANOVA were employed as our goal for this exploratory research was to generate a number of hypotheses for further testing and confirmation in a larger sample. Fourth, threshold selection for the fiber number of SC will lead to different network densities, which may influence the related results of SC (Kong L.Y. et al., 2021). In our study, the value was set to 3, which was the highest threshold that maintained the average size of the largest connected component at 90 across all subjects (Li et al., 2009; Wang et al., 2012; Zhang et al., 2015), meaning that the 90 brain regions in the network were all connected at this threshold value in the majority of the 157 subjects. Finally, our research mainly focused on gut microbial diversity and enterotypes because they are the most frequently used global parameters in the characterization of gut microbial community profiles. Other indices (e.g., relative abundance of the bacteria) derived from 16S sequencing analysis should be calculated to further examine the gut microbe–brain relationship in the future.

In conclusion, the results of this study provide the first empirical evidence that the gut microbiota can modulate brain network topological parameters and SC–FC couplings in young adulthood. Furthermore, some small-worldness of structural networks, nodal properties of functional networks, and SC–FC coupling of multiple brain regions can act as mediators of the effects of gut microbiota on cognition. These findings might expand existing biological knowledge concerning gut microbiota–brain–cognition relationships from the perspective of brain network topological properties. More generally, the study revealed novel insights, which are essential to provide the foundation for previously unexplored mechanisms in understanding cognitive impairment, particularly with respect to how brain connectivity participates in the complex crosstalk between gut microbiota and cognition.

## Data Availability Statement

The datasets presented in this study can be found in online repositories. The names of the repository/repositories and accession number(s) can be found below: National Center for Biotechnology Information (NCBI) BioProject database under accession number PRJNA793133.

## Ethics Statement

The studies involving human participants were reviewed and approved by the Ethics Committee of the First Affiliated Hospital of Anhui Medical University. The patients/participants provided their written informed consent to participate in this study. Written informed consent was obtained from the individual(s) for the publication of any potentially identifiable images or data included in this article.

## Author Contributions

SZ, JZ, and YY conceptualized and designed the study. SZ was responsible for conducting the analyses, preparing the first draft of the manuscript, and preparing the manuscript for submission. JZ and YY were responsible for obtaining funding for the study, supervising the analyses, and editing drafts of the manuscript. SZ, XX, QL, JC, SL, WZ, and HC were responsible for data collection and initial data preprocessing. All authors contributed to and approved the final manuscript.

## Conflict of Interest

The authors declare that the research was conducted in the absence of any commercial or financial relationships that could be construed as a potential conflict of interest.

## Publisher’s Note

All claims expressed in this article are solely those of the authors and do not necessarily represent those of their affiliated organizations, or those of the publisher, the editors and the reviewers. Any product that may be evaluated in this article, or claim that may be made by its manufacturer, is not guaranteed or endorsed by the publisher.

## Footnotes

1
http://www.fmrib.ox.ac.uk/fsl

2
http://trackvis.org/dtk

3
http://www.nitrc.org/projects/panda

4
http://rfmri.org/dpabi

5
http://www.nitrc.org/projects/Gretna

6
http://www.processmacro.org/

## References

[B1] Ahluwalia V. Wade J. B. Heuman D. M. Hammeke T. A. Sanyal A. J. Sterling R. K. (2014). Enhancement of functional connectivity, working memory and inhibitory control on multi-modal brain MR imaging with Rifaximin in Cirrhosis: implications for the gut-liver-brain axis. *Metab. Brain Dis.* 29 1017–1025. 10.1007/s11011-014-9507-6 24590688 PMC4155029

[B2] Arumugam M. Raes J. Pelletier E. Le Paslier D. Yamada T. Mende D. R. (2011). Enterotypes of the human gut microbiome. *Nature* 473 174–180. 10.1038/nature09944 21508958 PMC3728647

[B3] Bagga D. Aigner C. S. Reichert J. L. Cecchetto C. Fischmeister F. P. S. Holzer P. (2019). Influence of 4-week multi-strain probiotic administration on resting-state functional connectivity in healthy volunteers. *Eur. J. Nutr.* 58 1821–1827. 10.1007/s00394-018-1732-z 29850990 PMC6647073

[B4] Bassett D. S. Bullmore E. (2006). Small-world brain networks. *Neuroscientist* 12 512–523. 10.1177/1073858406293182 17079517

[B5] Baum G. L. Cui Z. Roalf D. R. Ciric R. Betzel R. F. Larsen B. (2020). Development of structure-function coupling in human brain networks during youth. *Proc. Natl. Acad. Sci. U S A* 117 771–778. 10.1073/pnas.1912034117 31874926 PMC6955327

[B6] Bernhardt B. C. Chen Z. He Y. Evans A. C. Bernasconi N. (2011). Graph-theoretical analysis reveals disrupted small-world organization of cortical thickness correlation networks in temporal lobe epilepsy. *Cereb. Cortex* 21 2147–2157. 10.1093/cercor/bhq291 21330467

[B7] Bullmore E. Sporns O. (2009). Complex brain networks: graph theoretical analysis of structural and functional systems. *Nat. Rev. Neurosci.* 10 186–198. 10.1038/nrn2575 19190637

[B8] Cai H. Wang C. Qian Y. Zhang S. Zhang C. Zhao W. (2021). Large-scale functional network connectivity mediate the associations of gut microbiota with sleep quality and executive functions. *Hum. Brain Mapp.* 42 3088–3101. 10.1002/hbm.25419 33739571 PMC8193524

[B9] Cao R. Wang X. Gao Y. Li T. Zhang H. Hussain W. (2020). Abnormal Anatomical Rich-Club Organization and Structural-Functional Coupling in Mild Cognitive Impairment and Alzheimer’s Disease. *Front. Neurol.* 11:53. 10.3389/fneur.2020.00053 32117016 PMC7013042

[B10] Caporaso J. G. Kuczynski J. Stombaugh J. Bittinger K. Bushman F. D. Costello E. K. (2010). QIIME allows analysis of high-throughput community sequencing data. *Nat. Methods* 7 335–336. 10.1038/nmeth0510-335 20383131 PMC3156573

[B11] Carlson A. L. Xia K. Azcarate-Peril M. A. Goldman B. D. Ahn M. Styner M. A. (2018). Infant Gut Microbiome Associated With Cognitive Development. *Biol. Psychiatry* 83 148–159. 10.1016/j.biopsych.2017.06.021 28793975 PMC5724966

[B12] Cieslik E. C. Mueller V. I. Eickhoff C. R. Langner R. Eickhoff S. B. (2015). Three key regions for supervisory attentional control: evidence from neuroimaging meta-analyses. *Neurosci. Biobehav. Rev.* 48 22–34. 10.1016/j.neubiorev.2014.11.003 25446951 PMC4272620

[B13] Collin G. Scholtens L. H. Kahn R. S. Hillegers M. H. J. van den Heuvel M. P. (2017). Affected Anatomical Rich Club and Structural-Functional Coupling in Young Offspring of Schizophrenia and Bipolar Disorder Patients. *Biol. Psychiatry* 82 746–755. 10.1016/j.biopsych.2017.06.013 28734460

[B14] Crespo-Facorro B. Roiz-Santianez R. Pelayo-Teran J. M. Gonzalez-Blanch C. Perez-Iglesias R. Gutierrez A. (2007). Caudate nucleus volume and its clinical and cognitive correlations in first episode schizophrenia. *Schizophr. Res.* 91 87–96. 10.1016/j.schres.2006.12.015 17306506

[B15] Faith D. P. (1992). Conservation evaluation and phylogenetic diversity. *Biol. Conserv. Biol. Conserv.* 61 1–10.

[B16] Falony G. Joossens M. Vieira-Silva S. Wang J. Darzi Y. Faust K. (2016). Population-level analysis of gut microbiome variation. *Science* 352 560–564. 10.1126/science.aad3503 27126039

[B17] Fan F. Liao X. Lei T. Zhao T. Xia M. Men W. (2021). Development of the default-mode network during childhood and adolescence: a longitudinal resting-state fMRI study. *Neuroimage* 226 117581. 10.1016/j.neuroimage.2020.117581 33221440

[B18] Franzosa E. A. Sirota-Madi A. Avila-Pacheco J. Fornelos N. Haiser H. J. Reinker S. (2019). Gut microbiome structure and metabolic activity in inflammatory bowel disease. *Nat. Microbiol.* 4 293–305. 10.1038/s41564-018-0306-4 30531976 PMC6342642

[B19] Gao W. Salzwedel A. P. Carlson A. L. Xia K. Azcarate-Peril M. A. Styner M. A. (2019). Gut microbiome and brain functional connectivity in infants-a preliminary study focusing on the amygdala. *Psychopharmacology* 236 1641–1651. 10.1007/s00213-018-5161-8 30604186 PMC6599471

[B20] Gareau M. G. (2016). Cognitive Function and the Microbiome. *Int. Rev. Neurobiol.* 131 227–246. 10.1016/bs.irn.2016.08.001 27793221

[B21] Gong G. L. He Y. Concha L. Lebel C. Gross D. W. Evans A. C. (2009). Mapping Anatomical Connectivity Patterns of Human Cerebral Cortex Using *In Vivo* Diffusion Tensor Imaging Tractography. *Cereb. Cortex* 19 524–536. 10.1093/cercor/bhn102 18567609 PMC2722790

[B22] Greicius M. D. Supekar K. Menon V. Dougherty R. F. (2009). Resting-State Functional Connectivity Reflects Structural Connectivity in the Default Mode Network. *Cereb. Cortex* 19 72–78. 10.1093/cercor/bhn059 18403396 PMC2605172

[B23] Groth-Marnat G. Baker S. (2003). Digit Span as a measure of everyday attention: a study of ecological validity. *Percept. Mot. Skills* 97 1209–1218. 10.2466/pms.2003.97.3f.1209 15002866

[B24] Hagmann P. Sporns O. Madan N. Cammoun L. Pienaar R. Wedeen V. J. (2010). White matter maturation reshapes structural connectivity in the late developing human brain. *Proc. Natl. Acad. Sci. U S A* 107 19067–19072. 10.1073/pnas.1009073107 20956328 PMC2973853

[B25] Hampson M. Driesen N. Roth J. K. Gore J. C. Constable R. T. (2010). Functional connectivity between task-positive and task-negative brain areas and its relation to working memory performance. *Magn. Reson. Imaging* 28 1051–1057. 10.1016/j.mri.2010.03.021 20409665 PMC2936669

[B26] Hayes A. F. (2009). Beyond Baron and Kenny: statistical Mediation Analysis in the New Millennium. *Commun. Monograph.* 76 408–420. 10.1080/03637750903310360

[B27] He Y. Dagher A. Chen Z. Charil A. Zijdenbos A. Worsley K. (2009). Impaired small-world efficiency in structural cortical networks in multiple sclerosis associated with white matter lesion load. *Brain* 132(Pt 12), 3366–3379. 10.1093/brain/awp089 19439423 PMC2792366

[B28] Hearne L. J. Lin H. Y. Sanz-Leon P. Tseng W. I. Gau S. S. Roberts J. A. (2019). ADHD symptoms map onto noise-driven structure-function decoupling between hub and peripheral brain regions. *Mol. Psychiatr.* 26 4036–4045. 10.1038/s41380-019-0554-6 31666679

[B29] Herlin B. Navarro V. Dupont S. (2021). The temporal pole: from anatomy to function-A literature appraisal. *J. Chem. Neuroanat.* 113:101925. 10.1016/j.jchemneu.2021.101925 33582250

[B30] Honey C. J. Sporns O. Cammoun L. Gigandet X. Thiran J. P. Meuli R. (2009). Predicting human resting-state functional connectivity from structural connectivity. *Proc. Natl. Acad. Sci. U. S A* 106 2035–2040. 10.1073/pnas.0811168106 19188601 PMC2634800

[B31] Honey C. J. Thivierge J. P. Sporns O. (2010). Can structure predict function in the human brain? *Neuroimage* 52 766–776. 10.1016/j.neuroimage.2010.01.071 20116438

[B32] Jenkins A. C. (2019). Rethinking Cognitive Load: a Default-Mode Network Perspective. *Trends Cogn. Sci.* 23 531–533. 10.1016/j.tics.2019.04.008 31176585

[B33] Johnson K. V. Foster K. R. (2018). Why does the microbiome affect behaviour? *Nat. Rev. Microbiol.* 16 647–655. 10.1038/s41579-018-0014-3 29691482

[B34] Kano M. Grinsvall C. Ran Q. Dupont P. Morishita J. Muratsubaki T. (2020). Resting state functional connectivity of the pain matrix and default mode network in irritable bowel syndrome: a graph theoretical analysis. *Sci. Rep.* 10:11015. 10.1038/s41598-020-67048-9 32620938 PMC7335204

[B35] Kaufman J. N. Ross T. J. Stein E. A. Garavan H. (2003). Cingulate hypoactivity in cocaine users during a GO-NOGO task as revealed by event-related functional magnetic resonance imaging. *J. Neurosci.* 23 7839–7843. 10.1523/JNEUROSCI.23-21-07839.2003 12944513 PMC6740597

[B36] Kelly A. M. Uddin L. Q. Biswal B. B. Castellanos F. X. Milham M. P. (2008). Competition between functional brain networks mediates behavioral variability. *Neuroimage* 39 527–537. 10.1016/j.neuroimage.2007.08.008 17919929

[B37] Kelsey C. M. Prescott S. McCulloch J. A. Trinchieri G. Valladares T. L. Dreisbach C. (2021). Gut microbiota composition is associated with newborn functional brain connectivity and behavioral temperament. *Brain Behav. Immun.* 91 472–486. 10.1016/j.bbi.2020.11.003 33157257

[B38] Keylock C. J. (2005). Simpson diversity and the Shannon–Wiener index as special cases of a generalized entropy. *Oikos* 109 203–207.

[B39] Kim B. R. Shin J. Guevarra R. Lee J. H. Kim D. W. Seol K. H. (2017). Deciphering Diversity Indices for a Better Understanding of Microbial Communities. *J. Microbiol. Biotechnol.* 27 2089–2093. 10.4014/jmb.1709.09027 29032640

[B40] Kong L. Y. Huang Y. Y. Lei B. Y. Ke P. F. Li H. H. Zhou J. (2021). Divergent Alterations of Structural-Functional Connectivity Couplings in First-episode and Chronic Schizophrenia Patients. *Neuroscience* 460 1–12. 10.1016/j.neuroscience.2021.02.008 33588002

[B41] Kong N. Gao C. Xu M. Gao X. (2021). Changes in the anterior cingulate cortex in Crohn’s disease: a neuroimaging perspective. *Brain Behav.* 11:e02003. 10.1002/brb3.2003 33314765 PMC7994700

[B42] Koubiyr I. Besson P. Deloire M. Charre-Morin J. Saubusse A. Tourdias T. (2019). Dynamic modular-level alterations of structural-functional coupling in clinically isolated syndrome. *Brain* 142 3428–3439. 10.1093/brain/awz270 31504228

[B43] Koubiyr I. Deloire M. Brochet B. Besson P. Charre-Morin J. Saubusse A. (2021). Structural constraints of functional connectivity drive cognitive impairment in the early stages of multiple sclerosis. *Mult. Scler.* 27 559–567. 10.1177/1352458520971807 33283582

[B44] Lackner J. M. Jaccard J. Keefer L. Brenner D. M. Firth R. S. Gudleski G. D. (2018). Improvement in Gastrointestinal Symptoms After Cognitive Behavior Therapy for Refractory Irritable Bowel Syndrome. *Gastroenterology* 155 47–57. 10.1053/j.gastro.2018.03.063 29702118 PMC6035059

[B45] Lei D. Li K. M. Li L. J. Chen F. Q. Huang X. Q. Lui S. (2015). Disrupted Functional Brain Connectome in Patients with Posttraumatic Stress Disorder. *Radiology* 276 818–827. 10.1148/radiol.15141700 25848901

[B46] Lemos L. N. Fulthorpe R. R. Triplett E. W. Roesch L. F. (2011). Rethinking microbial diversity analysis in the high throughput sequencing era. *J. Microbiol. Methods* 86 42–51. 10.1016/j.mimet.2011.03.014 21457733

[B47] Leng X. Xiang J. Yang Y. Yu T. Qi X. Zhang X. (2020). Frequency-specific changes in the default mode network in patients with cingulate gyrus epilepsy. *Hum Brain Mapp* 41 2447–2459. 10.1002/hbm.24956 32096905 PMC7268086

[B48] Li Y. H. Liu Y. Li J. Qin W. Li K. C. Yu C. S. (2009). Brain Anatomical Network and Intelligence. *Plos Computational Biology* 5:1000395. e100039510.1371/journal.pcbi.1000395 10.1371/journal.pcbi.1000395 PMC2683575 19492086

[B49] Liao X. Vasilakos A. V. He Y. (2017). Small-world human brain networks: perspectives and challenges. *Neurosci. Biobehav. Rev.* 77 286–300. 10.1016/j.neubiorev.2017.03.018 28389343

[B50] Lin Y. H. Young I. M. Conner A. K. Glenn C. A. Chakraborty A. R. Nix C. E. (2020). Anatomy and White Matter Connections of the Inferior Temporal Gyrus. *World Neurosurg.* 143 e656–e666. 10.1016/j.wneu.2020.08.058 32798785

[B51] Liu P. Jia X. Z. Chen Y. Yu Y. Zhang K. Lin Y. J. (2021). Gut microbiota interacts with intrinsic brain activity of patients with amnestic mild cognitive impairment. *CNS Neurosci. Ther.* 27 163–173. 10.1111/cns.13451 32929861 PMC7816203

[B52] Medaglia J. D. Huang W. Karuza E. A. Kelkar A. Thompson-Schill S. L. Ribeiro A. (2018). Functional Alignment with Anatomical Networks is Associated with Cognitive Flexibility. *Nat. Hum. Behav.* 2 156–164. 10.1038/s41562-017-0260-9 30498789 PMC6258039

[B53] Muller N. C. J. Konrad B. N. Kohn N. Munoz-Lopez M. Czisch M. Fernandez G. (2018). Hippocampal-caudate nucleus interactions support exceptional memory performance. *Brain Struct. Funct.* 223 1379–1389. 10.1007/s00429-017-1556-2 29138923 PMC5869896

[B54] Owen A. M. McMillan K. M. Laird A. R. Bullmore E. (2005). N-back working memory paradigm: a meta-analysis of normative functional neuroimaging studies. *Hum Brain Mapp* 25 46–59. 10.1002/hbm.20131 15846822 PMC6871745

[B55] Palop J. J. Mucke L. (2016). Network abnormalities and interneuron dysfunction in Alzheimer disease. *Nat. Rev. Neurosci.* 17 777–792. 10.1038/nrn.2016.141 27829687 PMC8162106

[B56] Park H. J. Friston K. (2013). Structural and functional brain networks: from connections to cognition. *Science* 342:1238411. 10.1126/science.1238411 24179229

[B57] Peng X. Wu X. Gong R. Yang R. Wang X. Zhu W. (2021). Sub-regional anterior cingulate cortex functional connectivity revealed default network subsystem dysfunction in patients with major depressive disorder. *Psychol. Med.* 51 1687–1695. 10.1017/S0033291720000434 32151293

[B58] Pereira J. B. Mijalkov M. Kakaei E. Mecocci P. Vellas B. Tsolaki M. (2016). Disrupted Network Topology in Patients with Stable and Progressive Mild Cognitive Impairment and Alzheimer’s Disease. *Cereb. Cortex* 26 3476–3493. 10.1093/cercor/bhw128 27178195 PMC4961019

[B59] Qian S. Zhang Z. Li B. Sun G. (2015). Functional-structural degeneration in dorsal and ventral attention systems for Alzheimer’s disease, amnestic mild cognitive impairment. *Brain Imaging Behav.* 9 790–800. 10.1007/s11682-014-9336-6 25452158

[B60] Raichle M. E. (2015). The brain’s default mode network. *Annu. Rev. Neurosci.* 38 433–447. 10.1146/annurev-neuro-071013-014030 25938726

[B61] Raichle M. E. MacLeod A. M. Snyder A. Z. Powers W. J. Gusnard D. A. (2001). A default mode of brain function. *Proc. Natl. Acad. Sci. U S A* 98 676–682. 10.1073/pnas.98.2.676 11209064 PMC14647

[B62] Rubinov M. Sporns O. (2010). Complex network measures of brain connectivity: uses and interpretations. *Neuroimage* 52 1059–1069. 10.1016/j.neuroimage.2009.10.003 19819337

[B63] Saji N. Murotani K. Hisada T. Tsuduki T. Sugimoto T. Kimura A. (2019). The relationship between the gut microbiome and mild cognitive impairment in patients without dementia: a cross-sectional study conducted in Japan. *Sci. Rep.* 9:19227. 10.1038/s41598-019-55851-y 31852995 PMC6920432

[B64] Sarkar A. Harty S. Lehto S. M. Moeller A. H. Dinan T. G. Dunbar R. I. M. (2018). The Microbiome in Psychology and Cognitive Neuroscience. *Trends Cogn. Sci.* 22 611–636. 10.1016/j.tics.2018.04.006 29907531

[B65] Schloss P. D. Westcott S. L. Ryabin T. Hall J. R. Hartmann M. Hollister E. B. (2009). Introducing mothur: open-source, platform-independent, community-supported software for describing and comparing microbial communities. *Appl. Environ. Microbiol.* 75 7537–7541. 10.1128/AEM.01541-09 19801464 PMC2786419

[B66] Seghier M. L. (2013). The angular gyrus: multiple functions and multiple subdivisions. *Neuroscientist* 19 43–61. 10.1177/1073858412440596 22547530 PMC4107834

[B67] Sherwin E. Bordenstein S. R. Quinn J. L. Dinan T. G. Cryan J. F. (2019). Microbiota and the social brain. *Science* 366:eaar2016. 10.1126/science.aar2016 31672864

[B68] Smallwood J. Bernhardt B. C. Leech R. Bzdok D. Jefferies E. Margulies D. S. (2021). The default mode network in cognition: a topographical perspective. *Nat. Rev. Neurosci.* 22 503–513. 10.1038/s41583-021-00474-4 34226715

[B69] Song K. Li J. Zhu Y. Ren F. Cao L. Huang Z. G. (2021). Altered Small-World Functional Network Topology in Patients with Optic Neuritis: a Resting-State fMRI Study. *Dis. Markers* 2021:9948751. 10.1155/2021/9948751 34221189 PMC8219459

[B70] Suarez L. E. Markello R. D. Betzel R. F. Misic B. (2020). Linking Structure and Function in Macroscale Brain Networks. *Trends Cogn. Sci.* 24 302–315. 10.1016/j.tics.2020.01.008 32160567

[B71] Sun Y. Dai Z. Li J. Collinson S. L. Sim K. (2017). Modular-level alterations of structure-function coupling in schizophrenia connectome. *Hum. Brain Mapp.* 38 2008–2025. 10.1002/hbm.23501 28032370 PMC6867028

[B72] Suo X. Lei D. Li K. Chen F. Li F. Li L. (2015). Disrupted brain network topology in pediatric posttraumatic stress disorder: a resting-state fMRI study. *Hum. Brain Mapp.* 36 3677–3686. 10.1002/hbm.22871 26096541 PMC6869652

[B73] Thomann A. K. Reindl W. Wustenberg T. Kmuche D. Ebert M. P. Szabo K. (2019). Aberrant brain structural large-scale connectome in Crohn’s disease. *Neurogastroenterol. Motil.* 31:e13593. 10.1111/nmo.13593 30983094

[B74] Tillisch K. Labus J. Kilpatrick L. Jiang Z. Stains J. Ebrat B. (2013). Consumption of Fermented Milk Product With Probiotic Modulates Brain Activity. *Gastroenterology* 144 1394.e–1401.e. 10.1053/j.gastro.2013.02.043 23474283 PMC3839572

[B75] Tu W. Ma Z. Ma Y. Dopfel D. Zhang N. (2021). Suppressing Anterior Cingulate Cortex Modulates Default Mode Network and Behavior in Awake Rats. *Cereb. Cortex* 31 312–323. 10.1093/cercor/bhaa227 32820327 PMC7727348

[B76] Turkiewicz J. Bhatt R. R. Wang H. Vora P. Krause B. Sauk J. S. (2021). Altered brain structural connectivity in patients with longstanding gut inflammation is correlated with psychological symptoms and disease duration. *Neuroimage Clin.* 30:102613. 10.1016/j.nicl.2021.102613 33823388 PMC8050027

[B77] Tzourio-Mazoyer N. Landeau B. Papathanassiou D. Crivello F. Etard O. Delcroix N. (2002). Automated anatomical labeling of activations in SPM using a macroscopic anatomical parcellation of the MNI MRI single-subject brain. *Neuroimage* 15 273–289. 10.1006/nimg.2001.0978 11771995

[B78] Vatansever D. Menon D. K. Manktelow A. E. Sahakian B. J. Stamatakis E. A. (2015). Default mode network connectivity during task execution. *Neuroimage* 122 96–104. 10.1016/j.neuroimage.2015.07.053 26220743

[B79] Vieira-Silva S. Falony G. Darzi Y. Lima-Mendez G. Garcia Yunta R. Okuda S. (2016). Species-function relationships shape ecological properties of the human gut microbiome. *Nat. Microbiol.* 1:16088. 10.1038/nmicrobiol.2016.88 27573110

[B80] Wang J. Khosrowabadi R. Ng K. K. Hong Z. Chong J. S. X. Wang Y. (2018). Alterations in Brain Network Topology and Structural-Functional Connectome Coupling Relate to Cognitive Impairment. *Front. Aging Neurosci.* 10:404. 10.3389/fnagi.2018.00404 30618711 PMC6300727

[B81] Wang J. Wang L. Zang Y. Yang H. Tang H. Gong Q. (2009). Parcellation-dependent small-world brain functional networks: a resting-state fMRI study. *Hum. Brain Mapp.* 30 1511–1523. 10.1002/hbm.20623 18649353 PMC6870680

[B82] Wang J. Wang X. Xia M. Liao X. Evans A. He Y. (2015). GRETNA: a graph theoretical network analysis toolbox for imaging connectomics. *Front. Hum. Neurosci.* 9:386. 10.3389/fnhum.2015.00386 26175682 PMC4485071

[B83] Wang Q. F. Su T. P. Zhou Y. Chou K. H. Chen I. Y. Jiang T. Z. (2012). Anatomical insights into disrupted small-world networks in schizophrenia. *Neuroimage* 59 1085–1093. 10.1016/j.neuroimage.2011.09.035 21963918

[B84] Wang Y. Nelissen N. Adamczuk K. De Weer A. S. Vandenbulcke M. Sunaert S. (2014). Reproducibility and robustness of graph measures of the associative-semantic network. *PLoS One* 9:e115215. 10.1371/journal.pone.0115215 25500823 PMC4264875

[B85] Wang Y. F. Zheng L. J. Liu Y. Ye Y. B. Luo S. Lu G. M. (2019). The gut microbiota-inflammation-brain axis in end-stage renal disease: perspectives from default mode network. *Theranostics* 9 8171–8181. 10.7150/thno.35387 31754388 PMC6857049

[B86] Watts D. J. Strogatz S. H. (1998). Collective dynamics of ‘small-world’ networks. *Nature* 393 440–442. 10.1038/30918 9623998

[B87] Zarkali A. McColgan P. Leyland L. A. Lees A. J. Rees G. Weil R. S. (2021). Organisational and neuromodulatory underpinnings of structural-functional connectivity decoupling in patients with Parkinson’s disease. *Commun. Biol.* 4 86. 10.1038/s42003-020-01622-9 33469150 PMC7815846

[B88] Zhang B. Wang H. E. Bai Y. M. Tsai S. J. Su T. P. Chen T. J. (2021). Inflammatory bowel disease is associated with higher dementia risk: a nationwide longitudinal study. *Gut* 70 85–91. 10.1136/gutjnl-2020-320789 32576641

[B89] Zhang J. Wang J. Wu Q. Kuang W. Huang X. He Y. (2011). Disrupted brain connectivity networks in drug-naive, first-episode major depressive disorder. *Biol. Psychiatry* 70 334–342. 10.1016/j.biopsych.2011.05.018 21791259

[B90] Zhang Z. Liao W. Chen H. Mantini D. Ding J. R. Xu Q. (2011). Altered functional-structural coupling of large-scale brain networks in idiopathic generalized epilepsy. *Brain* 134(Pt 10), 2912–2928. 10.1093/brain/awr223 21975588

[B91] Zhang R. Shao R. Xu G. Lu W. Zheng W. Miao Q. (2019). Aberrant brain structural-functional connectivity coupling in euthymic bipolar disorder. *Hum. Brain Mapp.* 40 3452–3463. 10.1002/hbm.24608 31282606 PMC6865442

[B92] Zhang R. Wei Q. Kang Z. Zalesky A. Li M. Xu Y. (2015). Disrupted brain anatomical connectivity in medication-naive patients with first-episode schizophrenia. *Brain Struct. Funct.* 220 1145–1159. 10.1007/s00429-014-0706-z 24449342

[B93] Zhang Y. Lin L. Lin C. P. Zhou Y. Chou K. H. Lo C. Y. (2012). Abnormal topological organization of structural brain networks in schizophrenia. *Schizophr. Res.* 141 109–118. 10.1016/j.schres.2012.08.021 22981811

[B94] Zheng L. J. Lin L. Zhong J. Zhang Z. Ye Y. B. Zhang X. Y. (2020). Gut dysbiosis-influence on amygdala-based functional activity in patients with end stage renal disease: a preliminary study. *Brain Imaging Behav.* 14 2731–2744. 10.1007/s11682-019-00223-3 32304020

[B95] Zhou D. Thompson W. K. Siegle G. (2009). MATLAB toolbox for functional connectivity. *Neuroimage* 47 1590–1607. 10.1016/j.neuroimage.2009.05.089 19520177 PMC2728136

[B96] Zimmermann J. Griffiths J. D. McIntosh A. R. (2018). Unique Mapping of Structural and Functional Connectivity on Cognition. *J. Neurosci.* 38 9658–9667. 10.1523/JNEUROSCI.0900-18.2018 30249801 PMC6595988

