# Supplementary material for: Brain Network Topology and Structural–Functional Connectivity Coupling Mediate the Association Between Gut Microbiota and Cognition
Source: Front Neurosci. 2022 Mar 29;16:814477. doi: 10.3389/fnins.2022.814477 (PMC9002058; doi:10.3389/fnins.2022.814477)
Supplement: Supplementary file 1 [file Table_1.DOC]

**Supplementary Materials**

**Supplementary methods**

**Cognition assessment**

**Working memory assessment**

The letter 3-back task was conducted on a computer to assess working memory using E-Prime 2.0 (<http://www.pstnet.com/eprime.cfm>) (Owen et al., 2005). During the task, each participant viewed a series of letters that were presented sequentially, and the presentation time of each letter stimulus was 200 ms with an interstimulus interval of 1800 ms. Participants were instructed to press a button on the right with their middle fingers if the letter that appeared on the screen was identical to the one presented 3 letters earlier and otherwise to press a button on the left with their index fingers. The task consisted of 60 trials. Before the formal test, participants were verbally instructed and had a practice test to ensure that they understood the task. The accuracy and mean reaction time of correct responses were used as the indices of working memory performance.

**Behavioral inhibition evaluation**

The Go/No-Go task was conducted on a computer to assess the ability of behavioral inhibition using E-Prime 2.0 (<http://www.pstnet.com/eprime.cfm>) (Kaufman JN et al., 2003). During the task, the letter X or Y was presented at a frequency of 1 Hz on the screen. In “Go” conditions, the current letter is different from the previous one and participants should respond quickly by pressing the button within 900 ms. In “No-Go” conditions (10% of all trials), the current letter is the same as the previous one and participants cannot press the button; if one presses the button, it would be counted as an error. The Go/No-Go task consisted of a practice test and a formal test. There were 20 trials (15 “Go” trials and 5 “No-Go” trials) in the practice test. If a participant responds correctly in 3 “No-Go” trials, he or she can shift to the formal test; otherwise, the participant needs to restart the practice test. The formal test was divided into two groups with 210 trials in each group and 30 s break between the two groups. It took about 12 min for the Go/No-Go task. The accuracy in “No-Go” conditions (Acc_No-Go) as well as the accuracy and mean reaction time of correct responses in “Go” conditions (Acc_Go and RT_Go) were used as the indices of task performance.

**Digit span tasks**

We also adopted digit span tasks to evaluate attention (Groth-Marnat G and S., 2003). All participants completed a digit span forward task followed by a digit span backward task. The former begins with a series of two digits orally presented to each participant continuing to a maximum of 13 digits. Participants were asked to verbally repeat the digits. There were two trials per digit series. All participants began with the first digit series (i.e., two digits), if repeated correctly, the participant continued to the next one, otherwise performed the second trial at the same digit series. The task was discontinued when the participant failed in the second trial. The span is defined as the maximum number of digits repeated by the participant. The digit span backward task followed the same procedure, except that participants verbally repeated the digits in reverse order.

**MRI data acquisition**

MRI scans were obtained using a 3.0-Tesla MR system (Discovery MR750w, General Electric, Milwaukee, WI, USA) with a 24-channel head coil. Earplugs were used to reduce scanner noise, and tight but comfortable foam padding was used to minimize head motion. High-resolution 3D T1-weighted structural images were acquired by employing a brain volume (BRAVO) sequence with the following parameters: repetition time (TR) = 8.5 ms; echo time (TE) = 3.2 ms; inversion time (TI) = 450 ms; flip angle = 12°; field of view (FOV) = 256 mm × 256 mm; matrix size = 256 × 256; slice thickness = 1 mm, no gap; 188 sagittal slices. Diffusion tensor imaging (DTI) data were acquired using a spin-echo single-shot echo planar imaging (SE-SS-EPI) sequence with the following parameters: TR = 10000 ms; TE = 74 ms; flip angle = 90°; FOV = 256 mm × 256 mm; matrix = 128 × 128; slice thickness = 3 mm without gap; 50 axial slices; 64 diffusion gradient directions (b = 1000 s/mm^2^) plus five b = 0 reference images. All images were visually inspected to ensure that only images without visible artifacts were included in subsequent analyses. Resting-state blood-oxygen-level-dependent (BOLD) fMRI data were acquired using a gradient-echo single-shot echo planar imaging (GRE-SS-EPI) sequence with the following parameters: TR = 2000 ms; TE = 30 ms; flip angle = 90°; FOV = 220 mm × 220 mm; matrix size = 64 × 64; slice thickness = 3 mm, slice gap = 1 mm; 35 interleaved axial slices; 185 volumes.

**DTI data preprocessing**

The software packages FMRIB Software Library (FSL, <http://www.fmrib.ox.ac.uk/fsl>)(Smith et al., 2004), Diffusion Toolkit (DTK, http://trackvis.org/dtk) and Pipeline for Analyzing braiN Diffusion imAges (PANDA, http://www. nitrc.org/projects/panda) (Cui et al., 2013) were used for the DTI preprocessing steps. Specifically, the diffusion-weighted images were first registered to a reference volume (i.e., the first b0 image) by using affine transformations to minimize distortions caused by the eddy currents and head motions. After skull-stripping, we estimated the 6 independent components of the diffusion tensor from which fractional anisotropy (FA) was calculated. Then a deterministic streamline tracking algorithm, i.e., Fiber Assignment by Continuous Tracking (FACT), was performed to obtain the whole-brain fiber tractography (Mori et al., 1999). The tracking procedure started from the deep white matter regions and terminated at a voxel with a turning angle greater than 45° or with an FA less than 0.2.

**Resting-state BOLD data preprocessing**

Resting-state BOLD data were preprocessed using Statistical Parametric Mapping software (SPM12, [http://www.fil.ion.ucl.ac.uk/spm](http://www.fil.ion.ucl.ac.uk/spm/software/spm8/)) and Data Processing & Analysis for Brain Imaging (DPABI, [http://rfmri.org/dpabi](http://rfmri.org/DPARSF)) (Yan et al., 2016). The first 10 volumes for each participant were discarded to allow the signal to reach equilibrium and the participants to adapt to the scanning noise. The remaining volumes were corrected for the acquisition time delay between slices. Then, realignment was performed to correct the motion between time points. Head motion parameters were computed by estimating the translation in each direction and the angular rotation on each axis for each volume. All participants’ BOLD data were within the defined motion thresholds (i.e., translational or rotational motion parameters less than 2 mm or 2°). We also calculated frame-wise displacement (FD), which indexes the volume-to-volume changes in head position. In the normalization step, individual structural images were firstly co-registered with the mean functional image; then the transformed structural images were segmented and normalized to the Montreal Neurological Institute (MNI) space using a high-level nonlinear warping algorithm, that is, the diffeomorphic anatomical registration through the exponentiated Lie algebra (DARTEL) technique (Ashburner, 2007). Finally, each filtered functional volume was spatially normalized to MNI space using the deformation parameters estimated during the above step and resampled into a 3-mm cubic voxel. After spatial normalization, all data sets were smoothed with a Gaussian kernel of 6 × 6 × 6 mm^3^ full-width at half maximum (FWHM).

**Structural network construction**

Because structural networks were constructed in native diffusion space, the AAL template in MNI space was needed to be transformed to individual native space. Briefly, individual structural images were firstly co-registered to their b0 images using a linear transformation. Then, the co-registered structural images were normalized to the MNI space using a nonlinear transformation. The derived transformation parameters were inverted and used to map the AAL template from MNI space to the native diffusion space (Gong et al., 2009). Then, the subject-specific AAL-ROIs were dilated 2-3 mm into the white matter to ensure that they were in contact with the fibers (Liao et al., 2011). In the native diffusion space, we calculated the number of fibers (with end-points located in both nodes during the fiber tracking) between any pairs of nodes, resulting in a 90 × 90 fiber number (FN) matrix for each subject. Two nodes (i.e., brain regions) were considered structurally connected if the FN between them was larger than or equal to three (Li et al., 2009; Wang et al., 2012; Zhang et al., 2015).

**Functional network construction**

Nodes and edges are two basic elements of a brain network. To define the nodes of brain networks, we used the automated anatomical labeling (AAL) template (Tzourio-Mazoyer et al., 2002) to parcellate the whole brain into 90 (45 for each hemisphere) cortical and subcortical regions of interest (ROIs). For each subject, the representative mean time series of each ROI was obtained by averaging the BOLD time series over all voxels within that region. To define the edges of functional brain networks, we computed the Pearson correlation coefficients between the regional mean time series of all possible pairs of nodes, resulting in a 90 × 90 correlation matrix for each subject. Finally, a Fisher z-transformation was applied to the FC matrices.

**Network topological metrics**

In the process of graph theoretical analysis, each FN matrix of structural network was thresholded and converted into a binary matrix, where the entry a_ij_ = 1 if the value of the FN between regions i and j was larger than or equal to three and a_ij_ = 0 otherwise.

For functional network, we applied a range of sparsity thresholds, which was defined as the ratio of the number of existing edges divided by the maximum possible number of edges in a network, to all correlation matrices. This approach guaranteed that all resultant networks would be comprised of the same number of edges, thereby enabling us to test the inter-group differences in relative network organization (Achard and Bullmore, 2007; He et al., 2009). In our study, a sparsity threshold range of 0.10 to 0.34 with an interval of 0.01 was employed according to several previous studies (Zhang et al., 2011; Lei et al., 2015; Suo et al., 2015). This thresholding strategy was determined such that the generated networks were estimable for the small-worldness and had sparse properties with the minimum possible number of spurious edges. Finally, each correlation matrix was thresholded and converted into a binary matrix, where the entry aij = 1 if the absolute value of the Pearson correlation coefficient between regions i and j was larger than the threshold and aij = 0 otherwise.

**Fecal samples collection and gut microbiota analysis**

Fecal samples were collected in sterilized tubes and stored immediately in a -80 ℃ freezer within 1 day before or after MRI examination. Microbial genome DNA was extracted from the fecal samples using a QIAamp DNA Stool Mini Kit (Qiagen Inc., Hilden, Germany). To construct the Polymerase Chain Reaction (PCR)-based 16S rRNA amplicon library for sequencing, PCR enrichment of the V4 hypervariable region of 16S rRNA gene was performed with the forward primer 515F (5’-GTGCCAGCMGCCGCGGTAA-3’) and reverse primer 806R (5’-GGACTACHVGGGTWTCTAAT-3’). The qualified amplicon mixture was then sequenced on the MiSeq platform with the PE250 sequencing strategy. Before the 16S rRNA data analysis, raw reads were filtered to remove adaptors and low-quality and ambiguous bases, and then paired-end reads were added to tags by the Fast Length Adjustment of Short reads program (FLASH, v1.2.11) (Magoc and Salzberg, 2011). The tags were clustered into operational taxonomic units (OTUs) with a cutoff value of 97% using UPARSE software (v9.1.13) (Edgar, 2013) and chimera sequences were compared with the Gold database using UCHIME (v4.2.40) (Edgar et al., 2011) to detect. Then, the representative sequence from each OTU cluster was obtained. These OTU representative sequences were taxonomically classified using Ribosomal Database Project (RDP) Classifier (v.2.2) (Wang et al., 2007) with a minimum confidence threshold of 0.8, and the training database was the Greengene Database (v201305) (DeSantis et al., 2006). The USEARCH_global (Edgar, 2010) was used to compare all tags back to OTU to get the OTU abundance statistics table of each sample.

Alpha diversity was assessed using the species diversity indices (including Shannon and Simpson indices) (Faith, 1992; Keylock, 2005), which were calculated by MOTHUR (v1.31.2) (Schloss et al., 2009) and QIIME (v1.8.0) (J Gregory Caporaso et al., 2010) at the OTU level. Shannon index measures the average degree of uncertainty in predicting where individual species chosen at random will belong, which increases as the number of species increases and as the distribution of individuals among the species becomes even (Lemos et al., 2011; Kim et al., 2017). Simpson index indicates the species dominance and reflects the probability of two individuals that belong to the same species being randomly chosen (Kim et al., 2017). Shannon and Simpson indices that reflect both species richness and species evenness. Sample enterotyping was performed based on OTU-derived genus abundance matrix as described in the original publication (Arumugam et al., 2011). Specifically, samples were clustered using Jensen-Shannon distance and partitioning around medoid (PAM) clustering. Calinski-Harabasz (CH) index was used to assess optimal number of clusters. The silhouette validation technique was utilized to assess the robustness of clusters.

**References**

Watts DJ, and SH., S. (1998). Collective dynamics of 'small-world' networks. Nature 393(6684), 440-442. doi: 10.1038/30918.


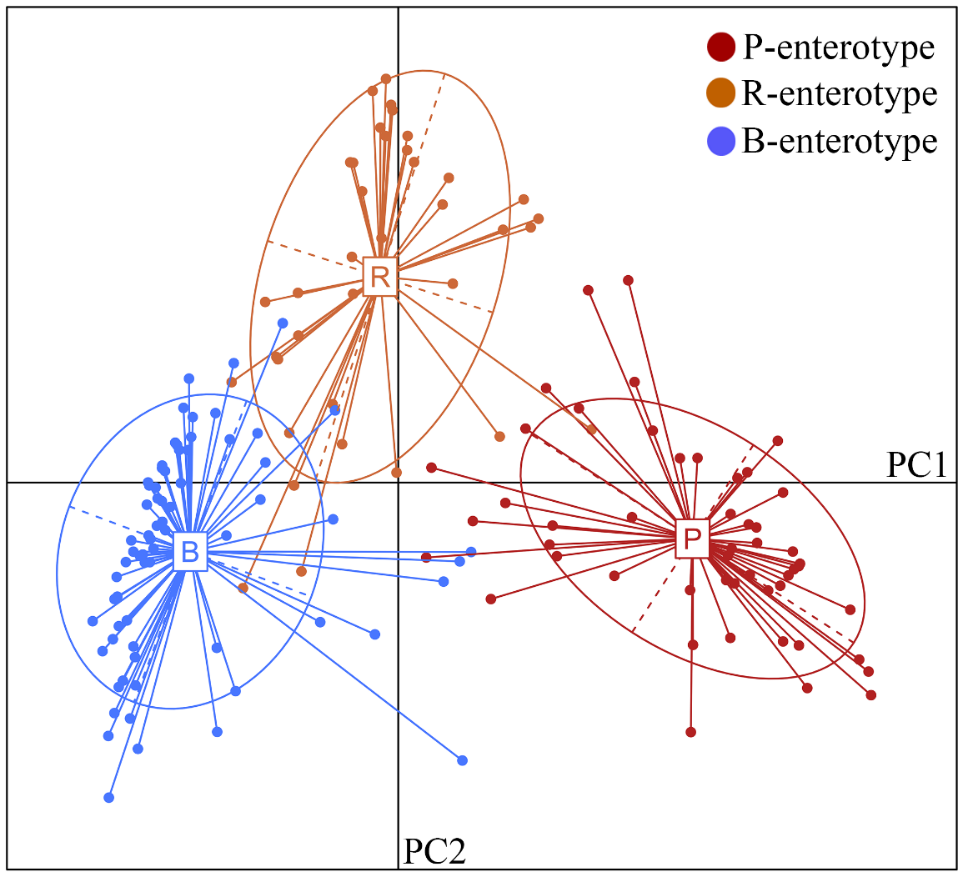


**Figure S2.** Jensen-Shannon distance and partitioning around medoid clustering is used to cluster all samples into three enterotypes. Abbreviations: PC, principal component; P, Prevotella; R, Ruminococcaceae; B, Bacteroides.

**Table S1 Demographic characteristics of the participants with the three enterotypes**

| Characteristics | P-enterotype | R-enterotype | B-enterotype | Statistics | *p* value |
| --- | --- | --- | --- | --- | --- |
| Number of subjects | 51 | 37 | 69 |  |  |
| Gender (female/male) | 24/27 | 21/16 | 32/37 | *χ*^2^ = 1.16 | 0.561^*^ |
| Age (years) | 22.55 ± 2.39 | 21.78 ± 2.27 | 22.45 ± 2.49 | *F* = 1.24 | 0.291^#^ |
| BMI (kg/m^2^) | 21.76 ± 3.95 | 21.25 ± 2.47 | 21.30 ± 2.93 | *F* = 0.39 | 0.680^#^ |
| Education (years) | 15.96 ± 1.93 | 15.54 ± 1.86 | 15.78 ± 1.96 | *F* = 0.51 | 0.602^#^ |
| FD (mm) | 0.13 ± 0.07 | 0.12 ± 0.04 | 0.12 ± 0.04 | *F* = 1.22 | 0.299^#^ |

Note: All values are expressed as mean ± standard deviation.
Abbreviations: B, bacteroides; BMI, body mass index; FD, frame-wise displacement; P, prevotella; R, ruminococcaceae.
^*^The p value was obtained by Pearson Chi-square test.
^#^The p value was obtained by one-way ANOVA.


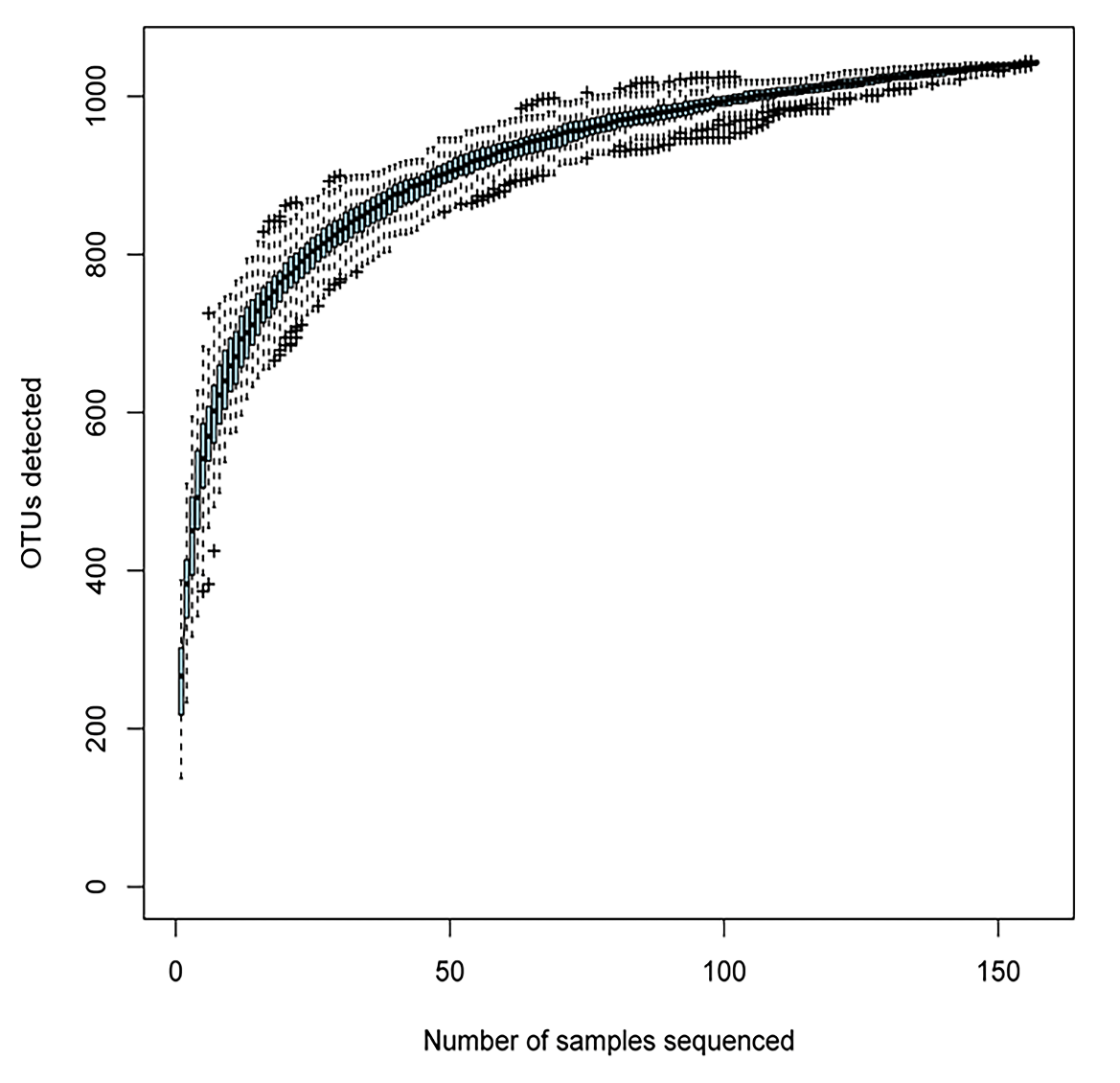


**Figure S3.** Species accumulation curves. The abscissa represents the number of samples, and the ordinate represents the number of OTUs (the number of detected species). Species accumulation curves reflect the influence of the number of samples on species diversity. The end of the curve tends to be flat, indicating that the sampling amount is sufficient. Abbreviations: OUT, operational taxonomic unit.


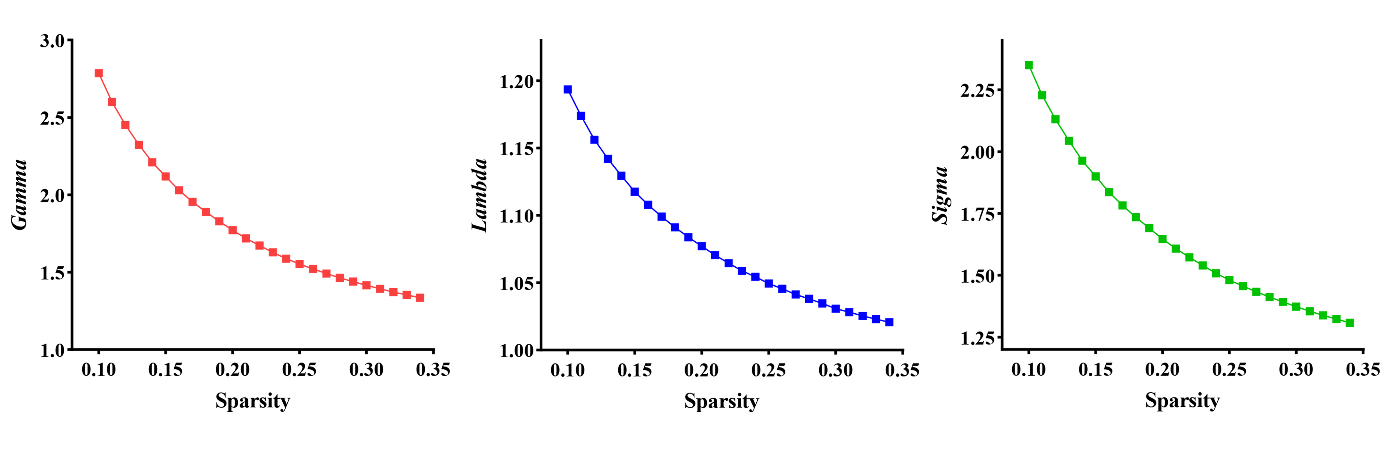


**Figure S4**. The small-world property metrics as a function of sparsity thresholds. In the defined threshold range, functional brain networks showed normalized clustering coefficients *Gamma* and small-worldness *Sigma* substantially greater than 1 and normalized characteristic path lengths *Lambda* approximately equal to 1, indicating that functional brain networks exhibited the typical feature of small-world topology. *Gamma*, normalized clustering coefficients; *Lambda*, normalized characteristic path lengths; *Sigma*, small-worldness.
